# Two-pore channel-2 controls calmodulin-dependent STIM1 inactivation

**DOI:** 10.1038/s41467-026-75158-7

**Published:** 2026-07-01

**Authors:** Subo Lee, Raphael Néré, Kyoung Sun Park, Vincent Jaquet, Nicolas Demaurex, Kyu-Sang Park

**Affiliations:** 1 Department of Physiology and Global Medical Science, Organelle Medicine Research Center, Yonsei University Wonju College of Medicine, Wonju, Republic of Korea; 2 Department of Cell Physiology and Metabolism, University of Geneva, Geneva, Switzerland

**Keywords:** Calcium signalling, Lysosomes, Endoplasmic reticulum

## Abstract

Lysosomal two-pore channels (TPC) trigger Ca^2+^ release from the endoplasmic reticulum (ER). The ensuing ER Ca^2+^ depletion activates STIM1-gated store-operated Ca^2+^ entry (SOCE) channels that sustain Ca^2+^ signals regulating fundamental cellular processes. How TPC channels and STIM1 integrate distinct intra and extracellular cues is unclear. Here, we show that TPC2 activation inhibits SOCE by enforcing rapid and persistent Ca^2+^-CaM-dependent inactivation of the STIM-Orai activating region (SOAR). The TPC2 activators NAADP and TPC2-A1-N abrogated SOCE in multiple cell lines and enhanced the slow Ca^2+^ dependent inactivation (SCDI) of STIM1-gated Orai1 channels. TPC2 engagement triggered lysosomal Ca^2+^ release and mobilized ER Ca^2+^ stores but prevented RFP-STIM1 recruitment to the TIRF plane by thapsigargin and disassembled RFP-STIM1 clusters forming after store depletion, preventing and acutely reversing SOCE. These effects persisted in STIM1 mutants truncated after the SOAR and were prevented by TPC2 genetic or pharmacological invalidation, Calmodulin (CaM) inhibition, and cytosolic Ca^2+^ chelation. We conclude that Ca^2+^ ions released by TPC2 channels on lysosomes regulate CaM-dependent STIM1 inactivation.

## Abstract (web summary)

Subo Lee and colleagues show that lysosomal two-pore channels (TPC) 2 unexpectedly block store-operated calcium entry by triggering calmodulin-dependent inactivation of STIM1. This previously unknown brake on calcium signaling suppresses T cell activation and microglial phagocytosis.

## Introduction

Lysosomes, once viewed as digestive compartments degrading and recycling cellular waste, are now recognized as critical Ca^2+^ signaling hubs that control cell fate and survival^1–6^. Lysosomes are Ca^2+^-sequestering organelles that can take up and release Ca²⁺ ions at membrane contact sites (MCSs) with other organelles, thereby contributing to the generation and propagation of local and global Ca^2+^ signals^7^, reviewed in ref. ^6^. The acidic luminal pH (4.5–5.0) of lysosomes, maintained by vacuolar V-type ATPases, facilitates the uptake of Ca^2+^ via the recently identified proton-activated lysosomal Ca²⁺ importer TMEM165^8,9^. MCSs forming between lysosomes and the endoplasmic reticulum (ER) enable direct and efficient Ca^2+^ transfer, accounting for the high luminal Ca^2+^ concentrations of lysosomes (~500–600 µM) and making these organelles the second-largest Ca^2+^ store in mammalian cells^10–13^. Conversely, lysosomal Ca²⁺ release at ER-lysosomes MCSs can trigger the release of Ca²⁺ from the larger ER stores, with individual lysosomal release events generating local transients that contribute to the propagation and amplification of global cellular Ca²⁺ signals^6,14–16^.

Two main ion channels contribute to lysosomal Ca^2+^ release: The Transient Receptor Potential Mucolipin 1 encoded by the *MCOLN1* gene and the Two Pore Segment Channel 2 (TPC2) encoded by the *TPCN2* gene. TPC2 is a dual-gated ion channel of late endosomes and lysosomes comprising two homologous domains that dimerize to form a functional “quasi-tetrameric” channel whose selectivity is agonist-dependent, switching between Na^+^ and Ca^2+^ based on the stimulus^17^. TPC2 is expressed in immune cells, participating in the release of pro-inflammatory cytokines, phagocytosis, and T-cell signaling^18^, and perturbations of TPC2-mediated calcium signals are positively or negatively associated with a wide range of inflammatory and neuro-degenerative human diseases^19,20^. Two main endogenous agonists, the second messenger nicotinic acid adenine dinucleotide phosphate (NAADP) and PI(3,5)P_2_, a phospholipid enriched in endolysosomal membranes, activate TPC2 to stimulate the release of Ca^2+^ and Na^+^, respectively^21–24^. This dual regulation enables TPC2 channels to dynamically switch between a Ca^2+^-permeable state and a Na^+^-selective state depending on the presence of NAADP and of PI(3,5)P_2_ on lysosomal membranes. NAADP, the most potent Ca^2+^-mobilizing second messenger initially identified in sea urchin eggs^25^, activates TPCs indirectly via two high-affinity NAADP-binding proteins, Jupiter microtubule associated homolog 2 (JPT2) and like-Sm protein 12 (LSM12) that form a complex with TPC2 channels^26–30^. In contrast, PI(3,5)P_2_ binds directly to a site on TPC2, inducing a structural change of a pore-lining helix that switches it to a Na^+^-selective channel^21,31^.

TPC2 coupling to ER Ca^2+^ channels amplifies NAADP Ca^2+^ signals by releasing Ca^2+^ from the ER and is therefore expected to trigger store-operated Ca^2+^ entry (SOCE), a fundamental physiological mechanism that replenishes ER Ca²⁺ levels following a decline in luminal Ca²⁺ concentration ([Ca^2+^]_ER_)^32^. SOCE is mediated by two ER-bound stromal interaction molecules (STIM1 and STIM2), which upon a decrease in [Ca^2+^]_ER_ levels sensed by luminal EF-hand domains unfold, oligomerize, and translocate to cortical ER structures juxtaposed to the plasma membrane (PM) to trap and gate Ca²⁺-permeable PM channels of the Orai and TRPC families^33–36^. Given the potential toxicity of Ca^2+^ ions, several intra and intermolecular mechanisms restrain STIM-Orai interactions to prevent inappropriate Ca^2+^ fluxes. STIM1 activates Orai1 channels via a short sequence of positively charged amino acid within STIM-Orai activating region (SOAR, aa 344–442) or channel activating domain (CAD, aa 342-448)^37,38^, which under resting conditions is masked by intramolecular interactions with an upstream coiled-coil region^39–43^ and with a distal inactivating domain (ID, aa 470-491)^44,45^. The later inactivation constraint is released by the SOCE-associated regulatory factor (SARAF), a single-pass ER protein^46,47^. SOCE termination requires both the restoration of basal [Ca^2+^]_ER_ levels and an increase in cytosolic Ca^2+^, a regulatory mechanism that prevents excessive Ca^2+^ influx^48–51^. This mechanism is mediated by calmodulin and by SARAF, which dissociates STIM1-Orai1 complexes and induces STIM de-oligomerization to promote the slow Ca²⁺-dependent inactivation (SCDI) of STIM1-gated Orai1 channels that has been well characterized at the electrophysiological level^46,47,52–54^.

Activation of ER Ca^2+^ release channels by NAADP is well documented, but the consequences of TPC2-mediated ER Ca^2+^ release on SOCE are unclear. TPC2 co-immunoprecipitates with STIM1 and Orai1 in MEG01 cells with depleted stores and TPC2 silencing mitigates SOCE, suggesting that TPC2 positively modulates SOCE via physical interactions with its molecular components^55^. However, membrane-permeant lysosomotropic drugs such as glycyl-L-phenylalanine 2-naphthylamide (GPN) and L-leucyl-L-leucine methyl ester (LLOMe), which induce lysosomal Ca^2+^ release, have unexpectedly been reported to inhibit SOCE^56^. To clarify the interactions between these two Ca^2+^ signaling circuits, we explored the effects of TPC2 engagement on STIM1/Orai1 molecular and functional interactions by ion imaging, electrophysiology, and FRET/TIRF microscopy. Using the synthetic agonist TPC2-A1-N, which mimics NAADP, we show that TPC2 activation induces ER Ca^2+^ release and Ca^2+^ depletion as efficiently as sarco(endo)plasmic reticulum Ca^2+^ ATPase (SERCA) inhibition. Unlike SERCA inhibition, TPC2 activation strongly suppresses STIM1 oligomerization, PM translocation, and SOCE across various cell types, inducing SCDI of STIM1-gated Orai1 channels. The deactivation of SOCE induced by TPC2 is mediated by calmodulin (CaM)-dependent disassembly of STIM1, driven by increased Ca²⁺ microdomains originating from lysosomes rather than the ER. The negative regulation of SOCE by TPC2 decreases T-cell activity and microglial phagocytosis, underscoring the critical role of TPC2 in modulating cellular Ca²⁺ signaling during immune responses.

## Results

### Activation of lysosomal TPC2 channels releases Ca^2+^ from ER stores without inducing SOCE

**Fig. 1:**
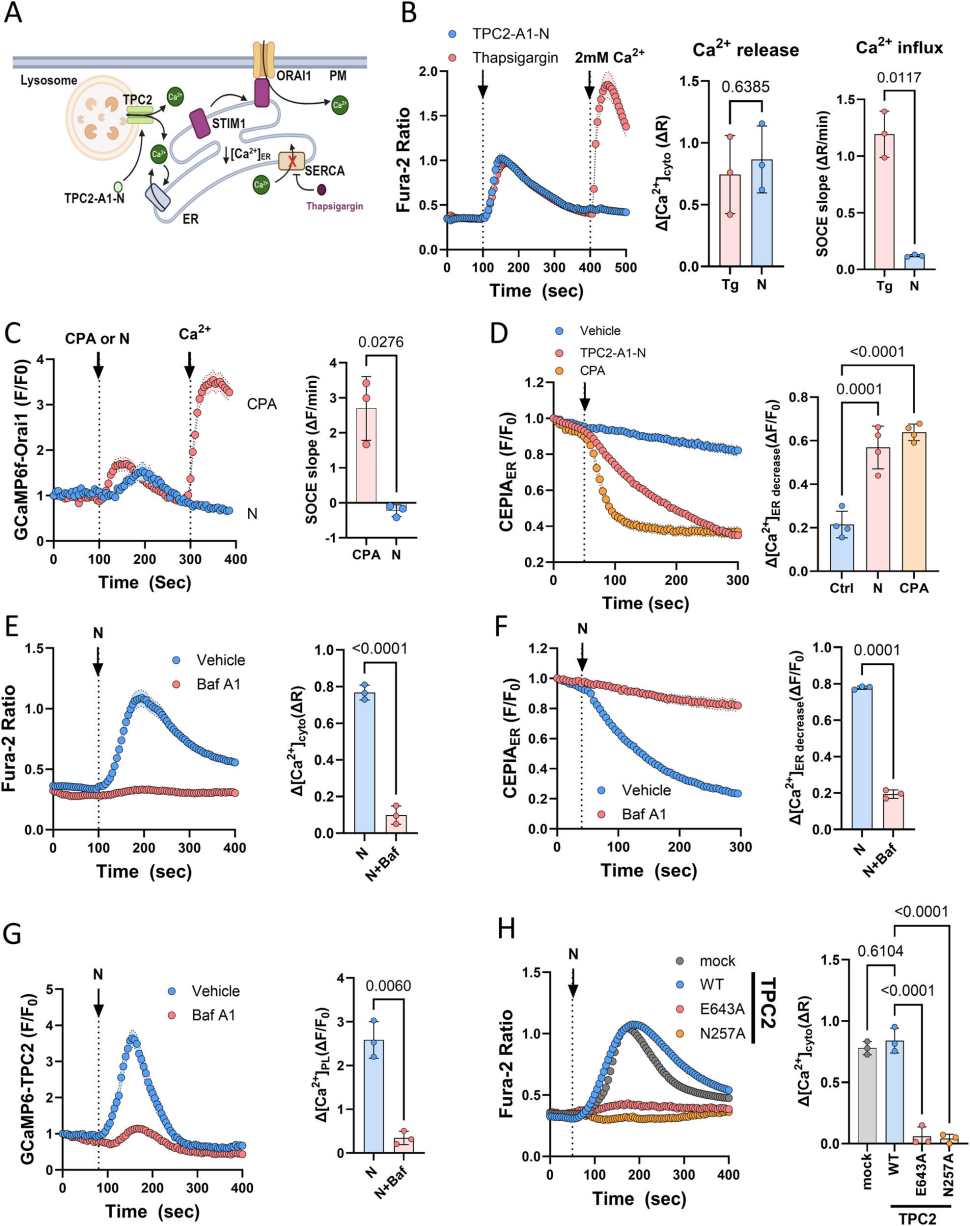
TPC2-A1-N releases ER Ca^2+^ without inducing SOCE. **A** Scheme of the calcium circuitry explored showing the molecular components targeted by TPC2-A1-N and by CPA/Tg and the mechanistic questions addressed in this study, created in BioRender. Park, K. (2026) https://BioRender.com/5b5y7po. **B** Left: Averaged Fura-2 ratio recordings of HEK-293 cells exposed to TPC2-A1-N (*N*, 20 μM, blue trace) or thapsigargin (Tg, 1 μM, red trace) in Ca^2+^-free media followed by 2 mM Ca^2+^. Right: quantification of [Ca^2+^]_cyt_ elevations resulting from Ca^2+^ release from intracellular stores and Ca^2+^ entry across membrane channels. **C** Left: Averaged GCaMP6f-Orai1 recordings of transfected HEK-293 cells exposed to TPC2-A1-N (*N*, 20 μM, blue trace) or cyclopiazomic acid (CPA, 10 μM, red trace) in Ca^2+^-free media followed by 2 mM Ca^2+^. Right: quantification of [Ca^2+^]_cyt_ elevations resulting from Ca^2+^ entry across membrane channels. **D** Averaged CEPIA_ER_ recordings (left) and quantification of [Ca^2+^]_ER_ decrease (right) evoked by DMSO (vehicle, green trace), TPC2-A1-N (*N*, 20 μM, blue trace) and CPA(CPA, 10 μM, red trace). Averaged Fura-2 ratio (**E**), CEPIA_ER_ (**F**), and GCaMP6-TPC2 (**G**) recordings (left) and quantification of [Ca^2+^]_cyt_, [Ca^2+^]_ER_, and [Ca^2+^]_peri-lyso_ changes (right) evoked by N in cells treated or not with bafilomycin A1 (0.1 µM) to inhibit V-ATPases and dissipate the pH gradient of acidic Ca^2+^ stores. (**H**) Averaged Fura-2 ratio recordings (left) and quantification of [Ca^2+^]_cyt_ elevations (right) evoked by TPC2-A1-N (N, 20 μM) in HEK-293 cells expressing pcDNA (gray trace), WT (blue trace) or dominant-negative TPC2 mutants (green and red traces). Data are presented as mean ± SD of *N* = 3 (**B**, **C**, **E**–**H**), or 4 (**D**) independent recordings from 127/115 cells for each group in the bar graph (the same applies below) (B) and 31/27 cells (**C**), two-tailed unpaired t test with Welch’s correction, 103/88/83 cells for each group (**D**) and 189/196, 121/100, and 58/55 cells (**E**–**G**), two-tailed unpaired t test with Welch’s correction, 114/187/144/165 cells (**H**), ordinary one-way ANOVA with Dunnett’s multiple comparisons test.

TPC2 channels trigger Ca^2+^ release from ER Ca^2+^ stores at the ER-lysosome interface^14^ and interact with STIM1 and Orai1 proteins to modulate SOCE^55^. To clarify how TPC2 channels impinge on the SOCE machinery, we used TPC2-A1-N, a small molecule TPC2 agonist that mimics the action of NAADP and favors TPC2 Ca^2+^ over Na^+^ permeability (Fig. 1A)^57,58^. When applied in Ca^2+^-free medium, TPC2-A1-N (20 μM) and the SERCA inhibitors, thapsigargin (Tg, 1 μM) or cyclopiazonic acid (CPA, 10 μM), evoked identical elevations in cytosolic Ca^2+^, [Ca^2+^]_cyt_ (Fig. 1B), as well as in subplasmalemmal Ca^2+^ measured by GCaMP6f-Orai1 (Fig. 1C) in HEK-293 cells by mobilizing Ca^2+^ from intracellular stores. However, unlike Tg or CPA, TPC2-A1-N failed to induce SOCE upon subsequent Ca^2+^ readmission (Fig. 1B, C). To verify that ER Ca^2+^ stores were depleted by TPC2-A1-N, we measured the changes in the free Ca^2+^ concentration within the ER lumen, [Ca^2+^]_ER_, with the genetically encoded indicator CEPIA_ER_. TPC2-A1-N decreased [Ca^2+^]_ER_ more slowly than CPA but to identical steady-state [Ca^2+^]_ER_ levels, indicating that ER stores were fully depleted within 4 min by the TPC2 agonist (Fig. 1D). Accordingly, CPA caused a minimal [Ca^2+^]_cyt_ elevation when applied after TPC2-A1-N in Ca^2+^-free medium (Supplementary Fig. 1A), and TPC2-A1-N failed to mobilize Ca^2+^ when applied after CPA (Supplementary Fig. 1B), indicating that intracellular stores were equally depleted by TPC2 engagement or SERCA inhibition. To confirm that TPC2-A1-N opens TPC2 channels, we measured peri-lysosomal Ca^2+^ elevations, [Ca^2+^]_PL_, with the genetically encoded indicator GCaMP6-TPC2. TPC2-A1-N caused a [Ca^2+^]_PL_ elevation that mirrored the [Ca^2+^]_cyt_ elevation and that persisted following ER stores depletion with CPA, indicating that it reflected lysosomal Ca^2+^ release (Supplementary Fig. 1C). These results further indicate that, in response to TPC2 agonists, the overall increase in [Ca^2+^]_cyt_ was primarily driven by Ca^2+^ release from the ER, the major intracellular Ca^2+^ store, whereas [Ca^2+^]_PL_, measured using GCaMP6-TPC2, more specifically reflected Ca^2+^ release from lysosomes (Supplementary Fig. 1D).

TPC2 channels are functionally coupled to ER Ca^2+^ release channels via Ca^2+^-induced Ca^2+^release (CICR), and local Ca^2+^ microdomains generated by TPC2 at ER-lysosomes contact sites can trigger the opening of either inositol trisphosphate receptors (IP_3_R) or ryanodine receptors (RyRs)^14,54,59,60^. To examine the role of local [Ca^2+^]_cyt_ elevations in CICR, cells were pretreated with a cell membrane-permeant Ca^2+^ chelator, BAPTA-AM (40 µM for 5 min), which suppresses [Ca^2+^]_cyt_ increases. Notably, BAPTA-AM prevented TPC2-A1-N-induced, but not CPA-induced, ER Ca^2+^ depletion, supporting a Ca^2+^-triggered mechanism for TPC2-mediated ER Ca^2+^ release (Supplementary Fig. 1E). We then used bafilomycin A1 (Baf A1), an inhibitor of V-ATPase, which depletes lysosomal Ca^2+^ pool (Supplementary Fig. 1E). Baf A1 (0.1 µM) abrogated changes in [Ca^2+^]_cyt_ (Fig. 1E), [Ca^2+^]_ER_ (Fig. 1F), and [Ca^2+^]_PL_ (Fig. 1G) evoked by TPC2-A1-N, further confirming the critical requirement of lysosomal Ca^2+^ for TPC2-induced Ca^2+^ changes.

We tested whether genetic manipulations of TPC2 impact TPC2-A1-N-mediated ER Ca^2+^ release. Expression of dominant-negative TPC2 mutants abrogated TPC2-A1-N-induced [Ca^2+^]_cyt_ elevations (Fig. 1H). Pharmacological inhibition of TPC2, using Tetrandrine (Tet, 3 μM) or Ned-19 (5 μM) (Supplementary Fig. 2A), also showed similar effects on TPC2-A1-N responses, abolishing [Ca^2+^]_cyt_ (Supplementary Fig. 2B), [Ca^2+^]_ER_ (Supplementary Fig. 2C), and [Ca^2+^]_PL_ (Supplementary Fig. 2D), while partial TPC2 silencing altered the kinetics but not the amplitude of the response (Supplementary Fig. 2E). Taken together, these data indicate that the opening of lysosomal TPC2 channels mobilizes Ca^2+^ from ER stores without inducing SOCE.

### TPC2 activation inhibits store-operated Ca^2+^ entry (SOCE)

**Fig. 2:**
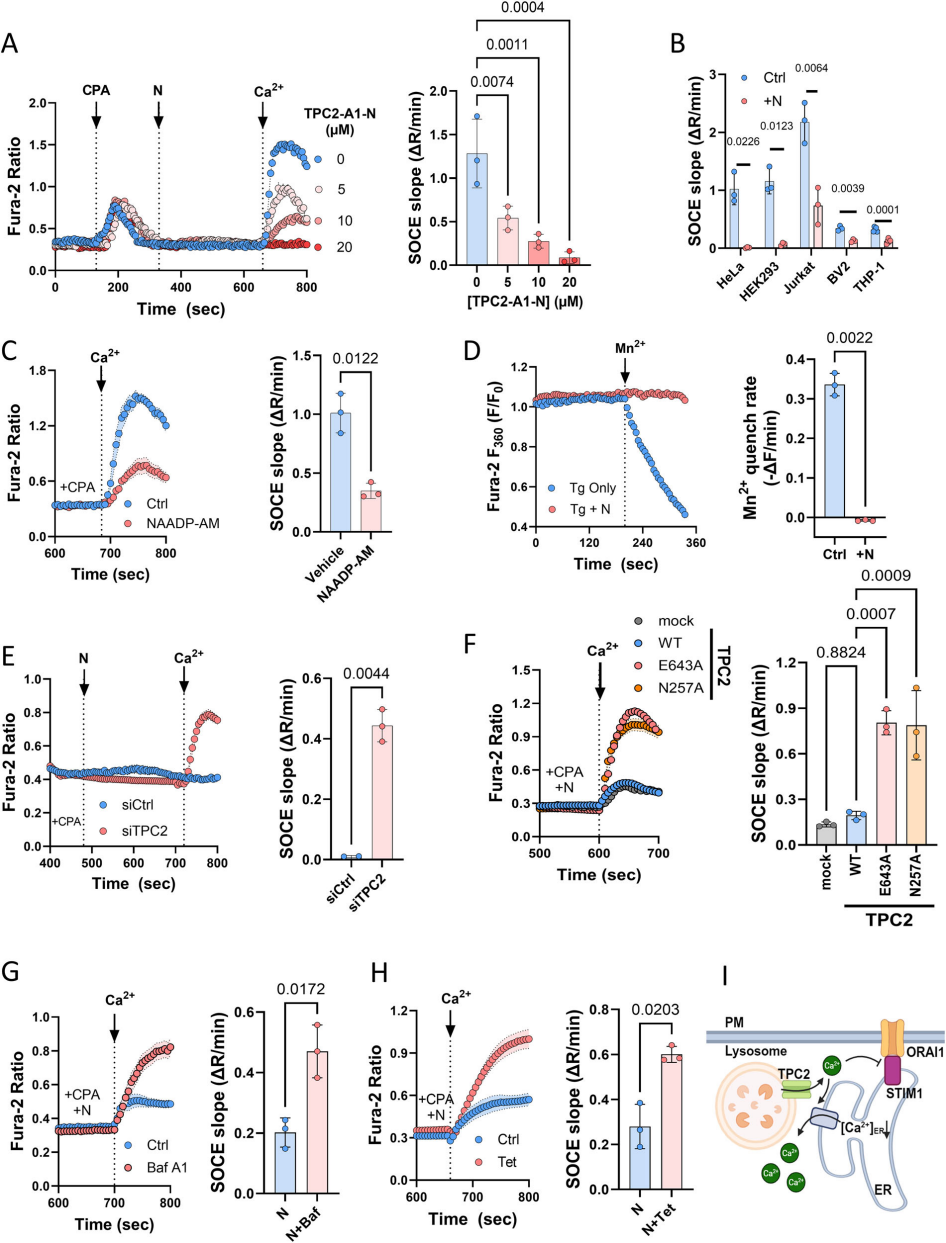
TPC2 activation inhibits store-operated Ca^2+^ entry (SOCE). **A** Averaged Fura-2 ratio recordings (left) and quantification of Ca^2+^ entry rates (right) in cells treated with CPA (10 µM) followed by increasing concentrations of TPC2-A1-N (N, 0-20 µM) applied for 5 min before Ca^2+^ readmission. **B** Quantification of Ca^2+^ entry rates in different cell lines exposed to CPA followed by 20 µM of N. **C** Averaged Fura-2 ratio recordings (left) and quantification of Ca^2+^ entry rates (right) in cells treated with CPA followed or not by 2 µM NAADP-AM for 5 min before Ca^2+^ readmission. **D** Averaged Fura-2 F_360_ recordings (left) and quantification of Mn^2+^ quenching rates (right) in Tg (1 μM)-treated cells exposed or not to 20 µM N before addition of 0.1 mM Mn^2+^. **E** Averaged Fura-2 ratio recordings (left) and quantification of Ca^2+^ entry rates (right) in cells transfected with scrambled and TPC2 targeting si-RNAs, exposed to CPA followed by 10 µM of N before Ca^2+^ readmission. **F** Averaged Fura-2 ratio recordings (left) and quantification of Ca^2+^ entry rates (right) in cells expressing empty vector (mock), WT, or dominant-negative TPC2 mutants (E643A, N257A), exposed to CPA followed by 10 µM of N before Ca^2+^ readmission. Averaged Fura-2 ratio recordings (left) and quantification of Ca^2+^ entry rates (right) in cells exposed or not to 100 nM Bafilomycin A1 (**G**) or 2 µM Tetrandrin (**H**), treated with CPA followed by 10 µM of N for 5 min before Ca^2+^ readmission. **I** Scheme showing the Ca^2+^ circuitry explored, created in BioRender. Park, K. (2026) https://BioRender.com/deh1otk. Data are mean ± SD of *N* = 3 (**A**–**H**) or 5 (THP1 in **B**) independent recordings from 107/102/104/88 cells for each group in the bar graph (**A**, from left to right), 108/112, 90/97, 112/116, 126/102, 120/145 cells (**B**, from left to right), 112/135 cells (**C**), 43/49 cells (**D**), 151/143 cells (**E**). 186/179/191 cells (**F**), 164/131 cells (**G**) and 166/128 cells (**H**). Ordinary one-way ANOVA with Dunnett’s multiple comparisons test (**A**, **B**, **F**) and two-tailed unpaired *t* test with Welch’s correction (**C**–**E**, **G**, **H**).

The lack of Ca^2+^ influx despite massive ER Ca^2+^ release observed upon TPC2 activation was unexpected, as ER Ca^2+^ depletion activates SOCE in nearly all cell types^32^ and TPC2 silencing attenuates SOCE in HEK-293 cells^55^. To clarify this paradox, we tested the impact of TPC2-A1-N on SOCE induced by SERCA inhibitors. TPC2-A1-N dose-dependently inhibited SOCE evoked by the readmission of Ca^2+^ to CPA (10 μM)-treated cells (Fig. 2A), an inhibition observed in five different cell lines: HeLa, HEK-293, Jurkat, BV2, and THP1 cells (Fig. 2B). Interestingly, TPC2-A1-P, a PI(3,5)P_2_ mimetic known to preferentially increase Na^+^ permeability of TPC2, did not inhibit SOCE by itself (Supplementary Fig. 3A, B), but augmented the suppressive effects of TPC2-A1-N on SOCE (Supplementary Fig. 3C-E). NAADP-AM (2 μM), a membrane-permeant version of the TPC2 natural agonist NAADP, also inhibited SOCE induced by CPA (Fig. 2C). Consistently, Tg-induced Mn^2+^ entry, reflecting the Ca^2+^ influx by ER Ca^2+^ depletion, was abrogated by TPC2-A1-N (10 μM) (Fig. 2D). Importantly, both TPC2 silencing and the expression of dominant-negative TPC2 mutants partially restored SOCE in TPC2-A1-N-treated cells (Fig. 2E, F), confirming the implication of TPC2 channels in this process. Accordingly, dissipation of lysosomal acidic pH with Baf A1 (0.1 µM) or pharmacological inhibition of TPC2 channels by Tet (3 μM) restored SOCE in TPC2-A1-N-treated cells (Fig. 2G, H), indicating that Ca^2+^ release from lysosomes, is responsible for TPC2-mediated SOCE regulation (Fig. 2I).

### TPC2 activation promotes slow Ca^2+^-dependent inactivation of STIM1-gated Orai1 channels

**Fig. 3:**
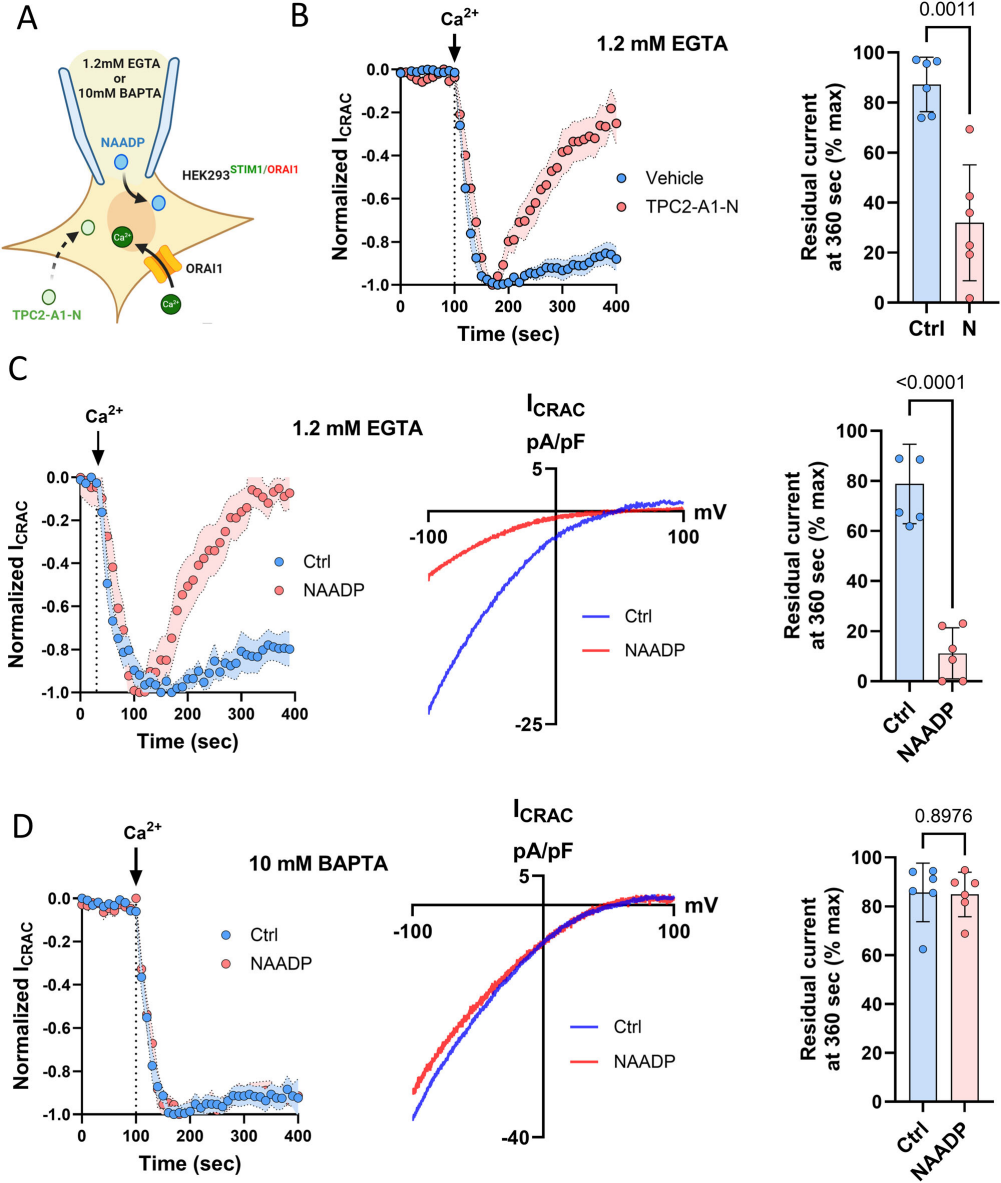
TPC2 activation enhances the SCDI of STIM1-gated Orai1 channels. **A** Sketch of the experimental conditions, created in BioRender. Park, K. (2026) https://BioRender.com/tihiv4p. **B** Normalized averaged whole-cell currents at −100 mV (left) and residual current densities at 360 sec (right) of HEK-293 cells expressing YFP-STIM1 and CFP-Orai1 (HEK^S1/O1^) recorded with pipette solutions containing 1.2 mM EGTA during addition of 2 mM Ca^2+^ to bath solutions containing or not 20 µM TPC2-A1-N. **C** Normalized averaged whole-cell currents (left), current-voltage (I-V) curves (middle), and residual current densities at 360 sec (right) of HEK^S1/O1^ cells recorded with pipette solutions containing 1.2 mM EGTA with or without 100 nM NAADP during addition of 2 mM Ca^2+^ to the bath solution. **D** Normalized averaged whole-cell currents (left) and residual current densities at 360 sec (right) of HEK^S1/O1^ cells recorded with pipette solutions containing 10 mM BAPTA with or without 100 nM NAADP during addition of 2 mM Ca^2+^ to the bath solution. Data are mean ± SEM of *N* = 6 recordings, two-tailed unpaired t test with Welch’s correction.

To gain insight into the mechanism of TPC2-mediated SOCE inhibition, we recorded Ca^2+^ release-activated Ca^2+^ current (I_CRAC_) in HEK-293 cells transiently expressing both STIM1 and Orai1 using the whole-cell configuration of the patch-clamp technique. Low amounts of Ca^2+^ buffers (1.2 mM EGTA) were included in pipette solutions to maximize the SCDI of STIM1-gated Orai1 channels (Fig. 3A). Addition of 2 mM Ca^2+^ to the bath solution evoked large inward-rectifying currents that decayed more rapidly in cells preincubated for 10 min with 20 (M TPC2-A1-N (Fig. 3B), indicating that TPC2 activation increases the SCDI of STIM1/Orai1-mediated I_CRAC_. TPC2-A1-N also delayed current activation and decreased the peak current density (Supplementary Fig. 4A), suggesting that the inactivation was already present when Ca^2+^ was added. Pipette application of 100 nM NAADP mimicked the effects of TPC2-A1-N, enhancing I_CRAC_ SCDI and decreasing the peak current density by 60% without impacting I_CRAC_ inward-rectifying voltage dependence (Fig. 3C and Supplementary Fig. 4B), whereas the same concentration of nicotinamide adenine dinucleotide phosphate (NADP) did not affect I_CRAC_ SCDI (Supplementary Fig. 4C). When NAADP was included in the pipette solution, the current decayed by ~90% in 4 min upon Ca^2+^ addition compared to ~20% in control conditions (Fig. 3C). Inclusion of 10 mM BAPTA in the patch pipette abolished both the I_CRAC_ SCDI triggered by NAADP and its associated reduction in peak current (Fig. 3D and S4D), confirming that the effects of NAADP are Ca^2+^-dependent. These data indicate that TPC2 activation by natural or artificial ligands enhances the SCDI of STIM1-gated Orai1 channels.

### TPC2 activation dissociates STIM1 oligomers and reverses STIM1 recruitment to the PM

**Fig. 4:**
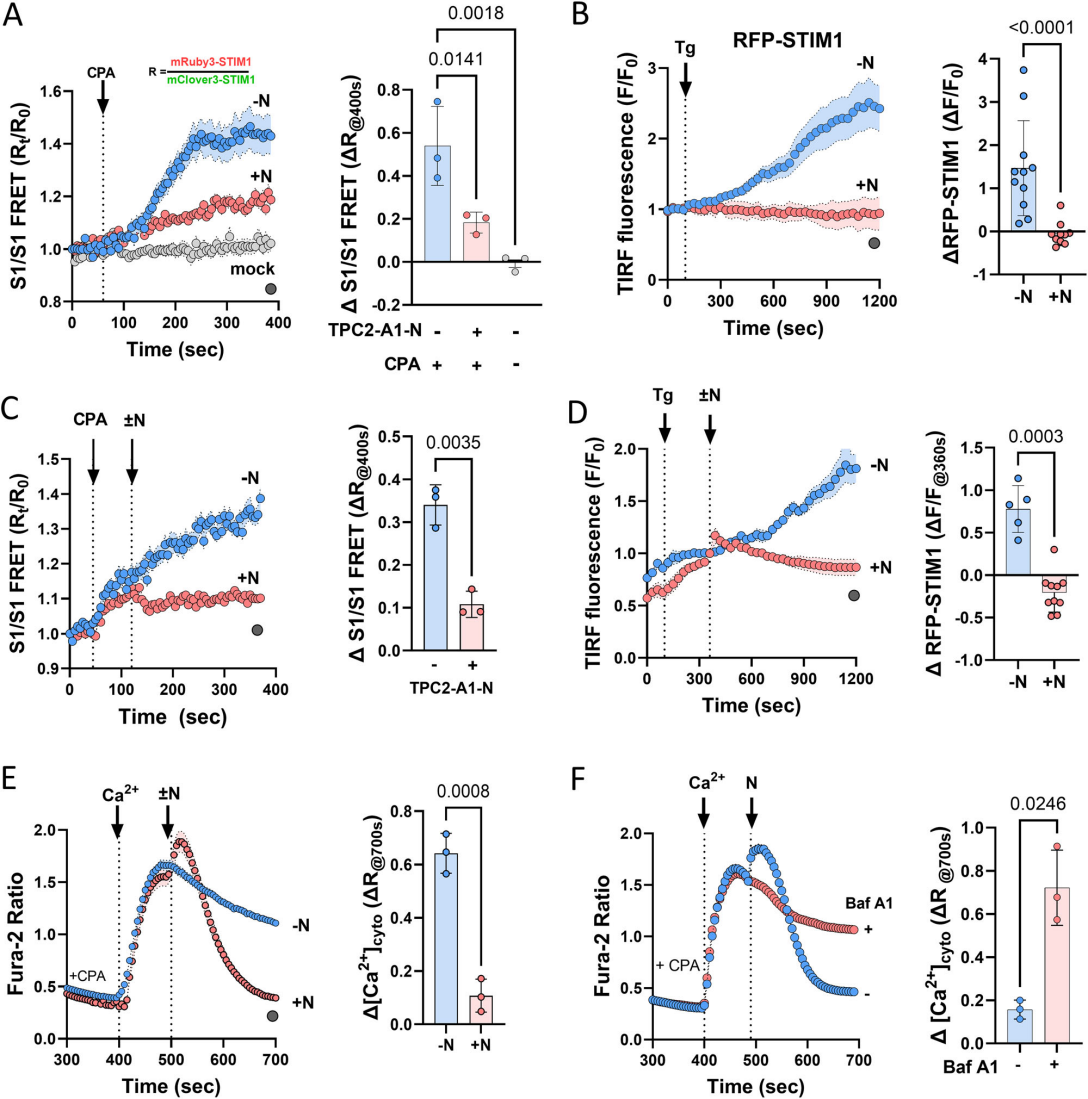
TPC2-A1-N dissociates STIM1 oligomers and reverses STIM1 PM translocation. **A** Changes in mRuby3-STIM1/mClover3-STIM1 FRET fluorescence evoked by CPA (10 µM) in cells pretreated or not with 20 µM TPC2-A1-N (*N*). Gray trace shows the FRET response of untreated cells. **B** Changes in RFP-STIM1 TIRF fluorescence evoked by 1 µM Tg in cells pretreated or not with *N*. **C** Changes in mRuby3-STIM1/mClover3-STIM1 FRET fluorescence evoked by *N* applied 100 sec after CPA addition. **D** Changes RFP-STIM1 TIRF fluorescence evoked by N applied 4 min after Tg addition. (**E**, **F**) Averaged Fura-2 ratio recordings (left) and quantification of SOCE decay rates (right) in CPA-treated cells exposed (red trace) or not (blue trace) to N (E) and bafilomycin A1 pretreated (red trace) or not (blue trace) (**F**) 100 sec after Ca^2+^readmission. Data are mean ± SD of *N* = 36/43/37 cells for each group in the bar graph (**A**, ordinary one-way ANOVA with Dunnett’s multiple comparisons test), 11/10 cells (**B**, two-tailed unpaired Mann-Whitney test), 31/22 cells (**C**), 5/10 cells (**D**), and *N* = 3 independent recordings from 252/201 (**E**),and 132/124 cells (**F**), two-tailed unpaired t test with Welch’s correction (**C**–**F**).

I_CRAC_ SCDI is a store-independent process driven by local [Ca^2+^]_cyt_ elevations that disrupt STIM1-ORA1 and STIM1-STIM1 interactions, thereby terminating SOCE^48–51^. To test whether TPC2 activation disassembles STIM1 oligomers, we measured the interactions between two fluorescent STIM1 constructs by FRET imaging. CPA (10 μM) increased the FRET between mRuby3-STIM1 and mClover3-STIM1, an increase prevented by TPC2-A1-N (20 μM) (Fig. 4A), indicating that TPC2 activation prevents the oligomerization of STIM1 triggered by store depletion. We then quantified the translocation of RFP-STIM1 into ER-PM clusters by TIRF imaging in HEK-293 cells lacking both STIM1 and STIM2 isoforms (DKO). TPC2-A1-N prevented cluster formation and the accumulation of RFP-STIM1 in the TIRF plane upon store depletion by Tg (1 μM) (Fig. 4B), indicating that TPC2 activation prevents the recruitment of STIM1 to the PM. Remarkably, when added after CPA or Tg, TPC2-A1-N decreased the FRET and TIRF signals, initiating STIM1 de-oligomerization and the retrieval of RFP-STIM1 from the TIRF plane (Fig. 4C, D). Accordingly, when TPC2-A1-N was added during or shortly after Ca^2+^ readmission, [Ca^2+^]_cyt_ rapidly returned to basal levels after SOCE initiation (Fig. 4E and S5A), consistent with a de-oligomerization and retrieval of PM-bound STIM1. As expected, inhibition of SOCE by TPC2-A1-N during or shortly after Ca^2+^ readmission were rescued by Baf A1 (0.1 μM)-induced lysosomal Ca^2+^ depletion (Fig. 4F and S5B). Furthermore, Baf A1 abolished actions of TPC2-A1-N on STIM1 de-oligomerization (Supplementary Fig. 5C, D) and the retrieval of RFP-STIM1 from surface (Supplementary Fig. 5E). These data indicate that TPC2 activation reverses the sequence of molecular interactions that drive STIM1 oligomerization, PM recruitment, and Orai1 gating.

### Calmodulin and STIM1 SOAR domain mediate TPC2-dependent SOCE inactivation

**Fig. 5:**
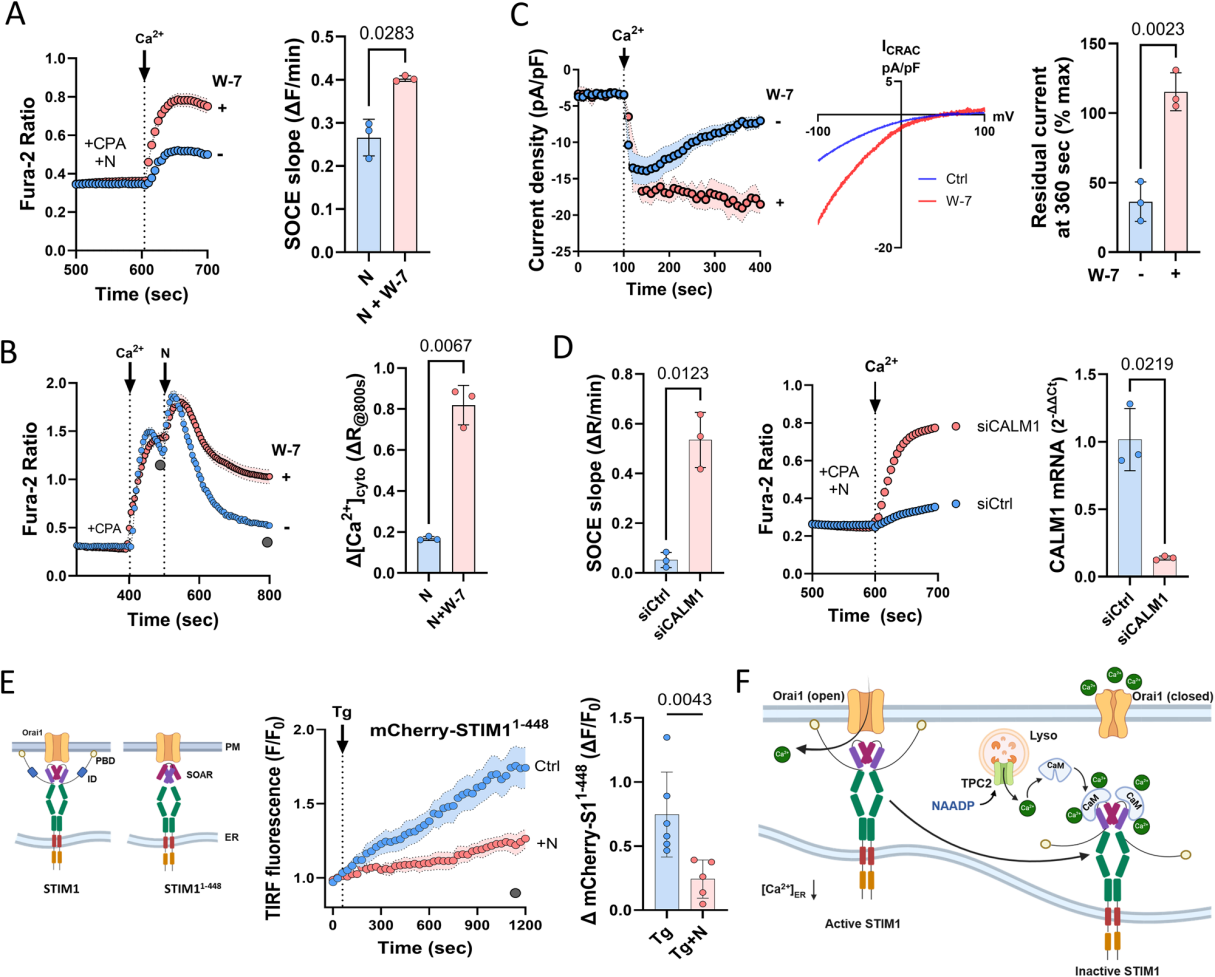
Calmodulin and the SOAR domain of STIM1 mediate TPC2-dependent STIM1 inactivation. **A** Averaged Fura-2 ratio recordings (left) and quantification of Ca^2+^ entry rates (right) in CPA-and TPC2-A1-N (N, 10 µM)-treated cells preincubated or not with W-7 (50 µM, 5 min). **B** Averaged Fura-2 ratio recordings (left) and quantification of steady-state [Ca^2+^]_cyt_ levels after Ca^2+^ readmission (right, dot indicates measurement time-point) in CPA-treated cells preincubated or not with W-7, exposed to N 100 sec after Ca^2+^ readmission. **C** Normalized averaged whole-cell currents (left), current-voltage (I-V) curves (middle), and residual current densities at 360 sec (right) recorded in HEK^S1/O1^ preincubated or not with W-7 (50 µM for 10 min) with pipette solutions containing 1.2 mM EGTA and 100 nM NAADP during addition of 2 mM Ca^2+^ to the bath solution. **D** CALM1 mRNA levels by RT-qPCR (left), averaged Fura-2 ratio recordings (middle) and quantification of [Ca^2+^]_cyt_ elevations (right) evoked by Ca^2+^ readmission in cells transfected with scrambled and CALM1 targeting si-RNAs. (**E**) Scheme of the truncated STIM1 construct showing the deleted domains, created in BioRender. Park, K. (2026) https://BioRender.com/bc64er8 and changes in mCherry-STIM1^1-448^ TIRF fluorescence evoked by Tg in cells pretreated or not with 20 µM N. **F** Scheme of TPC2 inactivate STIM1 Ca^2+^/CaM dependently, created in BioRender. Park, K. (2026) https://BioRender.com/0zsd6ig. Data are mean ± SD of *N* = 3 independent recordings from 146/129 cells, 146/129 cells and 181/211 cells for each group in the bar graph (**A**, **B**, **D**), of *N* = 3 /3 recordings (**C**), *N* = 6/5 cells (**E**), two-tailed unpaired *t* test with Welch’s correction (**A**–**D**), two-tailed unpaired Mann-Whitney test (**E**).

Our imaging and electrophysiological data show that TPC2 activation disrupts STIM1-Orai1 complexes, dissociates STIM1 oligomers, and facilitates I_CRAC_ SCDI. These effects phenocopy those of Ca^2+^-CaM binding to hydrophobic residues within the STIM1 Orai activating region (SOAR, residues 344–442)^52,53^ and of the inhibitory protein SARAF interacting with the SOAR of STIM1^46,47^. To test the involvement of CaM, we treated cells with the CaM inhibitor W-7. W-7 preincubation (50 µM for 10 min) mitigated TPC2-A1-N (10 μM)-mediated SOCE inhibition (Fig. 5A) and mitigated the rapid [Ca^2+^]_cyt_ decay evoked by TPC2-A1-N during Ca^2+^ readmission (Fig. 5B), consistent with CaM mediating the inhibitory effects of TPC2-A1-N. Importantly, SCDI of I_CRAC_ evoked by NAADP was abrogated by W-7 (Fig. 5C). Furthermore, siRNA-mediated CaM silencing rescued TPC2-induced SOCE inhibition, confirming that this effect is CaM-dependent (Fig. 5D). We then treated cells with BAPTA-AM, which relieved TPC2-A1-N inhibition of the CPA-induced FRET increase between mRuby3-STIM1 and mClover3-STIM1 (Supplementary Fig. 6A). Furthermore, BAPTA-AM prevented the acute inhibition of STIM1 oligomerization evoked by TPC2-A1-N observed in Fig. 4C (Supplementary Fig. 6B), indicating that cytosolic Ca^2+^ chelation mitigates the inhibitory effects of TPC2-A1-N on STIM1 oligomerization. These data indicate that activation of lysosomal TPC2 channels by endogenous or artificial ligands inhibits SOCE via a Ca^2+^-CaM-dependent process.

Ca^2+^-CaM binds to hydrophobic residues within SOAR while SARAF-SOAR interactions are controlled by the STIM1 inhibitory domain (ID, residues 470-491), a region that stabilizes STIM1 resting conformation and functions as a Ca^2+^ sensor for SCDI^44,61^. To test the involvement of STIM1 ID and SOAR domains in TPC2-mediated SOCE inhibition, we used a STIM1 truncation mutant lacking the ID domain but retaining the SOAR (STIM1^1-448^). Upon ER store depletion, STIM1^1-448^ adopts an extended conformation, oligomerizes, and translocate to the PM independently of Orai1 via interactions between its exposed SOAR with PM phosphoinositides^62^. Accordingly, mCherry-STIM1^1-448^ was efficiently recruited to the TIRF plane upon store depletion in DKO cells (Fig. 5E). TPC2-A1-N prevented mCherry-STIM1^1-448^ recruitment and reversed the increase in TIRF fluorescence initiated by Tg (Fig. 5E), indicating that the STIM1 ID domain is dispensable for TPC2 action. These data indicate that TPC2 activation alters the conformation of the SOAR domain of STIM1, preventing its interactions with PM lipids upon activation by store depletion, pointing to an involvement of Ca^2+^-bound CaM in TPC2-mediated STIM1 inactivation (Fig. 5F).

### TPC2-mediated SOCE inhibition modulates physiological Ca^2+^ signals in leukocytes

**Fig. 6:**
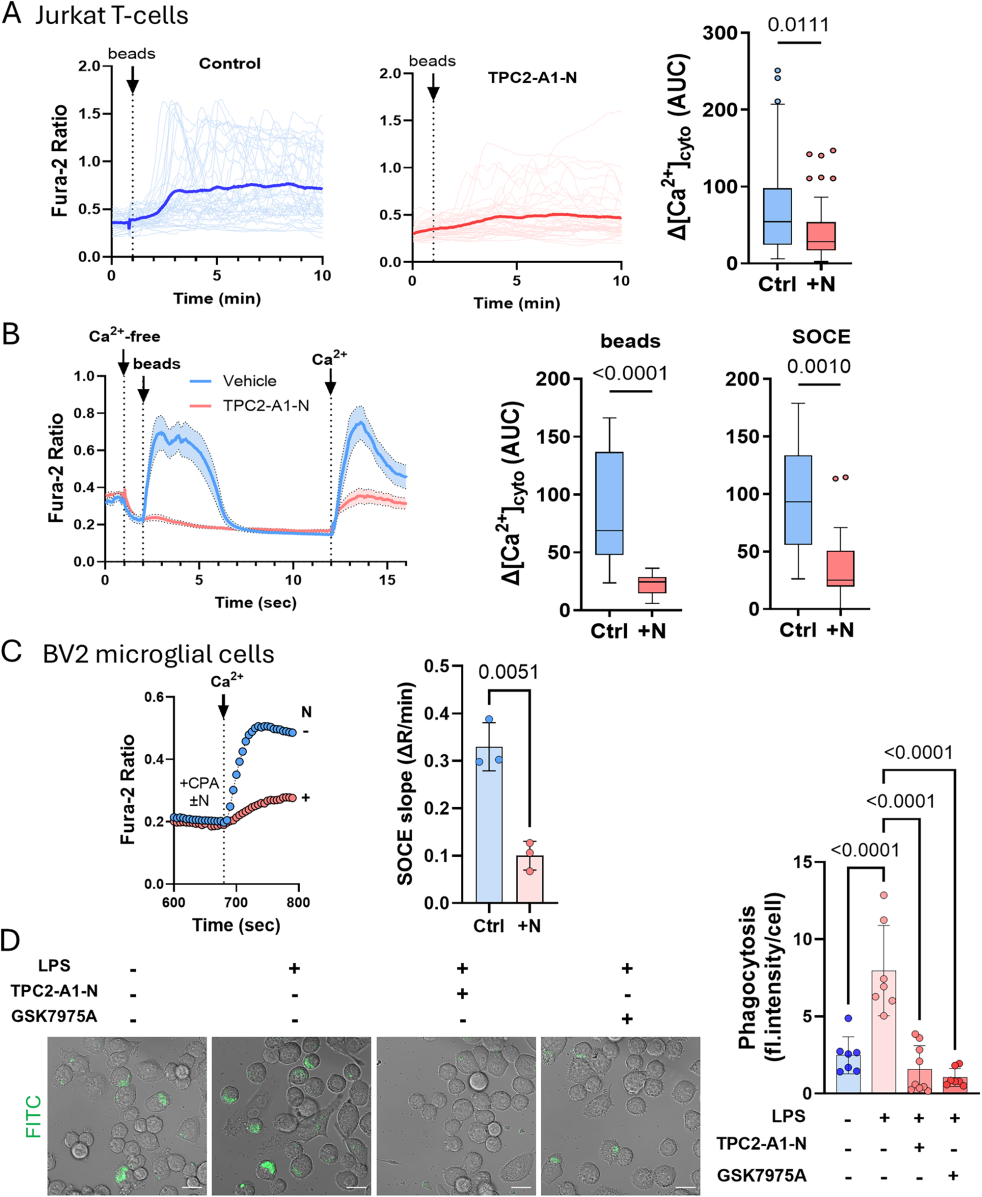
TPC2-A1-N inhibits physiological responses in leukocytes. **A** Individual (thin lines) and averaged (thick red line) Fura-2 responses evoked by CD3/CD28-coated beads in Jurkat T cells treated or not with 20 µM N in Ca^2+^-rich solution (left and middle panels, 40 cells per condition), and quantification of [Ca^2+^]_cyt_ responses during the 10 min recordings (right panels). Data are mean ± SEM of N = 69/55 cells from 3/2 recordings, two-tailed unpaired lognormal Welch’s *t* test. **B** Averaged Fura-2 responses evoked by CD3/CD28 beads followed by Ca^2+^ readmission in Jurkat T cells treated or not with 20 µM N in Ca^2+^-free solution (left), and quantification of [Ca^2+^]_cyt_ responses (right panels, AUC: 2-6 and 12-16 min, respectively). Data are mean ± SEM of N = 12/25 from 3/2 recordings, two-tailed unpaired Mann-Whitney test. **C** Averaged Fura-2 ratio recordings (left) and quantification of Ca^2+^ entry rates (right) in BV2 cells treated with CPA, exposed or not to 20 µM N for 5 min before Ca^2+^ readmission. Data are mean ± SD of *N* = 3 independent recordings from 226/217 cells for each group in the bar graph, two-tailed unpaired *t* test with Welch’s correction. **D** Fluorescence micrographs of BV2 microglial cells allowed to ingest fluorogenic E. coli particles for 20 min in the presence of GSK-7954A (2 µM) or N (20 µM) and quantification of phagocytosis efficiency (bottom). Scale bar = 20 μm. Data are mean ± SD of *N* = 7/7/9/7 recordings from 421/330/331/241 cells, two-tailed unpaired *t* test with Welch’s correction.

To assess how TPC2-mediated SOCE inhibition impacts physiological processes, we measured Ca^2+^ signals and effector responses in human leukocytes. Activation of Jurkat T-cells with CD3/CD28-coated beads evoked long-lasting Ca^2+^ elevations whose frequency and amplitude were reduced by preexposure to TPC2-A1-N (Fig. 6A). TPC2-A1-N preincubation (20 µM, 10 min) severely blunted [Ca^2+^]_cyt_ responses evoked by the beads in Ca^2+^-free medium and inhibited the response evoked by subsequent Ca^2+^ readmission (Fig. 6B). TPC2 activation thus inhibits both the Ca^2+^ release and influx component of the physiological response initiated by engagement of the T-cell receptor. In BV2 microglial cells, TPC2-A1-N also suppressed SOCE (Fig. 6C) and prevented the uptake of fluorescent bioparticles as efficiently as the CRAC channel inhibitor GSK-7975A (Fig. 6D). These data indicate that TPC2 activation suppresses physiological responses in T cell activation and macrophage phagocytosis, in part by inhibiting SOCE.

## Discussion

In this study, we show that activation of the lysosomal TPC2 channel by endogenous or artificial ligands inhibits SOCE by promoting the Ca^2+^-CaM-dependent inactivation of STIM1, thereby blunting physiological Ca^2+^ signals that control leukocytes effector functions. Using imaging and electrophysiology, we show that TPC2 activation by NAADP or by TPC2-A1-N inhibits SOCE and facilitates I_CRAC_ SCDI and identify Ca^2+^-CaM and the SOAR domain of STIM1 as the effector and target of this inhibition. Lysosomal Ca^2+^ release by TPC2 channels promoted ER Ca^2+^ release. However, TPC2 activation did not induce SOCE despite complete ER store depletion. Instead, TPC2 engagement triggered the dissociation of STIM1 oligomers, prevented and acutely reversed the translocation of STIM1 to ER-PM clusters, promoted SCDI and terminated SOCE. Using mutagenesis and TIRF microscopy, we further show that the inactivation of STIM1 by TPC2 is prevented by Ca^2+^ chelation and CaM inhibition but not by the deletion of STIM1 domains distal to SOAR.

**Fig. 7:**
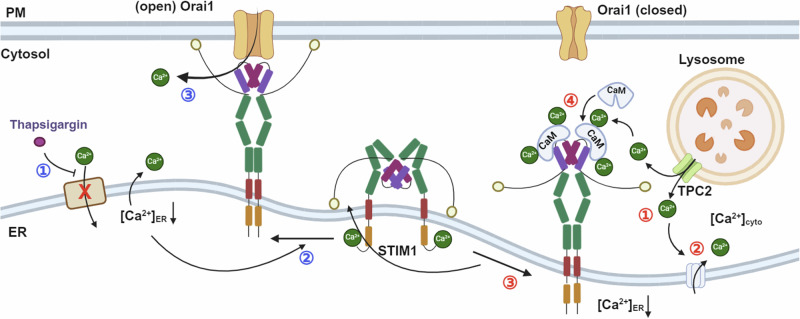
Proposed model. Left: STIM1 activation steps, numbered in blue. A [Ca^2+^]_ER_ drop induced by Tg (1) triggers the apposition of STIM1 luminal domains and TM helixes (2), exposing the SOAR and PBD that interacts with PM lipids. This conformational change enables STIM1 to trap Orai1 Ca^2+^ channels at the PM via its exposed SOAR (3). Right: Inactivation by TPC2, steps numbered in red. The opening of lysosomal TPC2 channels by NAADP generates [Ca^2+^]_cyt_ microdomains at ER-lysosome contact sites (1) that open RyR channels on the ER, decreasing [Ca^2+^]_ER_ and promoting STIM1 extension (3). In addition, NAADP-mediated lysosomal Ca^2+^ release activates CaM which in its Ca^2+^-bound form (Ca/CaM) binds to hydrophobic residues on the exposed SOAR (4). The SOAR-bound Ca/CaM acts as a shield that prevents productive interactions of the SOAR with Orai1 and with PM lipids, inactivating SOCE (5), created in BioRender. Park, K. (2026) https://BioRender.com/cahwca5.

Store depletion initiates a sequence of conformational changes that exposes STIM1 C-terminal polybasic domain (PBD) and SOAR, two domains that bind phosphoinositides^37,62^, enabling STIM1 to accumulate at ER-PM MCSs and to capture Orai1 channels by a diffusion trap mechanism^32^. The first step is the dissociation of Ca^2+^ ions from luminal STIM1 EF-hand domains, which elicits the dimerization of adjacent SAM domains^63^. The apposition of the two EF-SAM induces the zipping of the TM helixes, conveying the activation signal across the ER membrane to elicit molecular rearrangements in STIM1 cytosolic domains^64^. This releases an autoinhibitory clamp between the first coiled-coil domain and the SOAR, known as “CC1 clamp”^39–43^, promoting STIM1 extension, oligomerization, and exposing the PBD that binds to negatively charged PM lipids (Fig. 7). Full SOAR exposure additionally requires the release of another intramolecular clamp with a downstream inhibitory domain (CTID, residues 475–583), a step controlled by SARAF^44,46^. The individual steps rely on reversible changes in affinity between protein domains, and the whole sequence is reversed upon refilling of ER Ca^2+^ stores.

Several intrinsic and extrinsic negative regulatory factors maintain STIM1 in a folded, inactive conformation to ensure rapid and reversible activation while preventing the inappropriate engagement of SOCE. The importance of these mechanisms is highlighted by the deleterious effect of gain-of-function mutations in STIM1^65^. The critical activation step is the exposure of the SOAR, which binds to PM phosphoinositides via a short sequence of basic amino acids (_382_KKIKK)^39,62,66^ and to the Orai1 C-terminus via its apex residues^66–68^. Four intramolecular inhibitory mechanisms prevent the premature exposure of the SOAR in cells with replete stores^69^: 1) The EF-SAM maintain the luminal and TM domains separated in the Ca^2+^-bound state; 2) The CC1 clamp maintains STIM1 folded and positions the SOAR apex near the ER membrane; 3) hydrophobic and electrostatic interactions of two CC1 domains at the base of SOAR stabilize STIM1 dimers; and 4) electrostatic interactions between basic SOAR residues (_382_KIKKKR) and negatively charged ER lipids maintain the SOAR apex away from PM lipids and Orai1. Once these brakes are relieved, additional mechanism prevent excessive STIM1 activation. The CTID shields the SOAR and provides an activation constraint relieved by SARAF^44,46^, while a more distal EB-1 binding domain (_642_TRIP) traps STIM1 on microtubules, restricting PM cluster formation and promoting I_CRAC_ SCDI^70,71^. These inhibitory brakes collectively maintain STIM1 in a quiescent, responsive state and mutations that disrupt any of these restraints trigger spontaneous STIM1 activation^65^.

The use of the truncated STIM1^1-448^ mutant enabled us to narrow down the site of TPC2 inhibition. TPC2-A1-N inhibited STIM1^1-448^ PM translocation, excluding the involvement of PBD-mediated lipid binding and of microtubule trapping, both mediated by distal domains absent in the truncated mutant. Similarly, we can rule out that TPC2 is acting via the CTID or via SARAF to enforce the STIM1 inactive conformation. Our observation that TPC2 engagement prevents STIM1^1-448^ PM recruitment by Tg further rules out an involvement of the EF-SAM luminal domains, which are in the Ca^2+^-unbound apo state after irreversible store depletion with Tg. Instead, our data point to the involvement of Ca^2+^-bound CaM and of the SOAR of STIM1 in mediating TPC2 inhibitory effects. In store-depleted cells, TPC2 activity facilitated SCDI, dissociated STIM1 oligomers, and promoted the detachment of STIM1 from PM clusters, terminating SOCE. This phenocopies the effects of calmodulin activation by Ca^[2+ 52,53^ and indicates that TPC2 inhibitory effects involve the disruption of SOAR interactions with the PM and with Orai1.

Two prior studies analysed the molecular basis of Ca^2+^/CaM-mediated SOCE deactivation and I_CRAC_ SCDI^52,53^. They showed that Ca^2+^-bound CaM is recruited to activated STIM1 and binds to exposed hydrophobic residues within SOAR, in particular L390 and F391. The binding of Ca^2+^/CaM to SOAR competes with SOAR-Orai1 and with SOAR-PIP_2_ interactions, initiating the dissociation of STIM1-Orai1 complexes, the detachment of STIM1 from PM clusters, and the disassembly of STIM1 oligomers, a sequence of deactivation that underlies the SCDI of Orai1 channels. We report here that TPC2 agonists mimic the effects of Ca^2+^-bound CaM on STIM1 cluster formation, SCDI, and SOCE, and that their inhibitory effects are prevented by Ca^2+^ chelation and by CaM inhibition. This implicates Ca^2+^/CaM in the TPC2 inhibition. Given previous biochemical and genetic evidence that Ca^2+^/CaM binds to hydrophobic SOAR residues, the simplest mechanistic explanation for the TPC2-mediated SOCE inhibition is that Ca^2+^ ions released by TPC2 at the ER-lysosome interface recruit Ca^2+^/CaM to the exposed SOAR of vicinal STIM1 dimers, preventing STIM1 from interacting with Orai1 and PM lipids (Fig. 7).

The potency and specificity of Ca^2+^ elevations generated by lysosomal TPC2 channels in promoting the recruitment of Ca^2+^-bound CaM to the exposed SOAR of STIM1 is intriguing. Large Ca^2+^ microdomains are generated by the opening of IP_3_R on the ER without promoting Ca^2+^/CaM-dependent STIM1 inactivation. On the contrary, these Ca^2+^ release events are temporally associated with ER store depletion and ensuing STIM1 activation. Dantrolene prevented TPC2-mediated ER Ca^2+^ release but not TPC2-induced SOCE inhibition, indicating that the inhibitory Ca^2+^ signal originates from lysosomes and not from RyR channels. Several mechanisms could account for the opposite functional consequences of Ca^2+^ ions released from lysosomes or from ER channels. Lysosomes form MCSs with the ER, placing TPC2 channels in a prime location to detect STIM1 exposure. An attractive possibility is that the exposed SOAR interacts with negatively charged lipids on lysosomes via its stretch of lysine residues (_382_KKIKK)^39,62,66^. These residues stabilize the SOAR on the PM and ER membranes by binding to phosphoinositides and could play a similar role on lysosomal membranes as the SOAR switches orientation upon activation (Fig. 7). Lysosomes are enriched in PI(3,5)P_2_, the phosphoinositide with the strongest affinity for SOAR dimers in protein-lipid overlay assays^61^. This protein-lipid interaction would anchor the SOAR on lysosomes and facilitate the detection of TPC2 Ca^2+^ signals by lysosome-bound SOAR. Alternatively, the spatiotemporal pattern of Ca^2+^ microdomains generated by TPC2 might differ from those generated by the opening of IP_3_R channels to enable rapid and persistent Ca^2+^/CaM activation, and/or CaM could be pre-recruited to lysosome membranes and thereby preferentially respond to Ca^2+^ microdomains at the ER-lyso interface (Fig. 7). Regardless of the underlying mechanism, the potent and selective recruitment of Ca^2+^/CaM to SOAR by Ca^2+^ ions released by TPC2 channels enables lysosomes to negatively control SOCE during NAADP signaling.

We also demonstrate the physiological consequences of TPC2 engagement for leukocyte effector functions. In Jurkat T cells, TPC2-A1-N pretreatment prevented bead-evoked Ca^2+^ elevations in Ca^2+^-free medium and the subsequent Ca^2+^ entry upon Ca^2+^ readmission. This implies that lysosomes regulate Ca^2+^ release as well as entry in T cells. In BV2 microglial cells, TPC2-A1-N prevented the phagocytosis of bioparticles as efficiently as the Orai1 channel inhibitor GSK-7975A. This is congruent with previous findings from our lab and other implicating STIM1 and Orai1 in pro-phagocytic Ca^2+^ signals. We previously showed that STIM1 is recruited to phagosomes and promotes phagocytosis by generating periphagosomal [Ca^2+^]_cyt_ elevations that drive actin shedding and phagosome maturation in neutrophils^72^. By controlling STIM1 inactivation, TPC2 channels will imping on this pathway in two ways, by maintaining ER Ca^2+^ stores in a depleted state and by preventing interactions between STIM1 and Orai1 channels on phagosomes. This would efficiently prevent the generation of local Ca^2+^ elevations at ER-phagosomes MCSs by stopping Ca^2+^ fluxes across both ER-bound IP_3_R and phagosome-bound Orai1. We previously reported that activation of the lysosomal TRPML1 channel induces SOCE by promoting ER Ca^2+^ release, without exerting the SOCE inhibitory action of TPC2^10,73^. This indicates that the two lysosomal Ca^2+^ release channels are similarly coupled to ER Ca^2+^ release channels but differentially coupled to STIM1. TPC2 coimmunoprecipitates with STIM1^55^, suggesting that TPC2-mediated Ca^2+^ elevations could directly act on STIM1, but whether TRPLM1 interacts with STIM1 is not known. Alternatively, the two lysosomal channels could generate Ca^2+^ nanodomains of different spatiotemporal profiles, due to differences in Ca^2+^ permeability, ion fluxes, or locations at the ER-lysosome interface. Notably, these two channels are reported to produce opposite functional consequences: either activation of TRPML1 or inhibition of TPC2 ameliorates pathologies related to Alzheimer disease^74,75^. Further experiments will clarify how these two lysosomal channels interact with SOCE molecular components and whether the regulation of SOCE by lysosomal channels contributes to the pathogenesis of neuro-degenerative or -inflammatory diseases.

In summary, our findings reveal that the ER Ca^2+^-sensing protein STIM1 is negatively regulated by TPC2 channels. The local Ca^2+^ released by lysosomes not only triggers the release of vicinal ER Ca^2+^ release channels but also initiates the Ca^2+^-CaM-dependent inactivation of STIM1, thereby facilitating Orai1 channel inactivation. This mode of Ca^2+^-dependent inhibition likely involves the binding of Ca^2+^-CaM to hydrophobic residues within the SOAR domains of STIM1. We propose that TPC2 channels dynamically regulate STIM1 conformational changes to minimize PM Ca^2+^ fluxes during the coordinated mobilization of lysosomal and ER Ca^2+^ stores.

### Limitations of the study

We used a high dose of the artificial ligand of TPC2-A1-N to engage TPC2. This compound was recently reported to act independently of two-pore channels^76^. The effects of dominant-negative TPC2 mutants and of TPC2 silencing are not equivalent to the genetic ablation of TPC2, which remains to be tested. We cannot exclude a direct effect of TPC2 activation on Orai1 or on other SOCE modulatory proteins expressed in our cellular systems. Experiments in cells lacking these molecular players are required to clarify this point.

## Methods

### Cell culture

HEK-293 cells with triple knockout of IP_3_ receptors, IP_3_R1/2/3, were kindly provided by Prof. David Yule (Univ. Rochester)^77^. STIM1/2 double knockout and ORAI1/2/3 triple knockout cells were kindly provided by Prof. Mohamed Trebak (Univ of Pittsburg)^78^ and Barbara Niemeyer (Saarland University, Germany), respectively^79^. BV2 cells, a microglial cell line, were kindly provided by Prof. Yunjong Lee (Sungkyunkwan Univ., Korea). Other cells used in this study were obtained from ATCC (HEK-293, HeLa, and Jurkat cells) and the Korean Cell Line Bank (THP-1 cells). HEK-293 cells were cultured in Dulbecco’s modified Eagle’s medium (DMEM) (Sigma-Aldrich) supplemented with 10% fetal bovine serum (Hyclone) in 37 °C, 5% CO_2_ humidified incubator. Jurkat cells and BV2 cells

### Plasmids and transfection

G-GEPIA1-ER (#105012), GCaMP6-TPC2 (#240465), TPC2 mutants E643A (#135187) and N257A (#135186) were obtained from Addgene (Watertown, MA, USA). For FRET experiments, mRuby3-STIM1 and mClover3-STIM1 plasmids were kindly gifted from Prof. Chan Young Park (UNIST, Korea). RFP-STIM1 was created by gene synthesis, replacing YFP in YFP-STIM1 (kind gift from Anant Parekh) with RFP (GeneCust; Dudelange, Luxembourg)^80^. The RFP-STIM1 truncated mutant (1-448) was generated by gene editing. All plasmids were transfected using Lipofectamine® 2000 Transfection Reagent (Thermo Fisher Scientific) according to the manufacturer’s instructions.

### Electrophysiology

HEK-293 cells were transiently transfected with YFP-STIM1 and CFP-Orai1 in a ratio of 3:1 for 24 h at 37 °C with 5% CO_2_. After trypsin digestion, transfected cells were plated onto 35 mm dishes with culture medium, and then I_CRAC_ currents were recorded using the whole-cell configuration at room temperature. Pipettes were pulled from 1.5 mm thin-wall glass capillaries (GC150TF, Harvard Apparatus) using a Sutter P-97 micropipette puller (World Precision Instruments) to obtain a resistance between 2-3 MW. Currents were recorded with Pulse/Pulsefit software (HEKA Electronik, Lambrecht, Germany), using the EPC10 amplifier (Axon Instruments, Molecular Devices). Voltage ramps of 100 ms were applied from –100 to +100 mV after a 50 ms voltage step to −100 mV from a holding potential of 0 mV every 10 s. The standard bath solution contained 140 mM NaCl, 1 mM MgCl_2_, 2 mM CaCl_2_ and 10 mM HEPES (300–310 mOsm, pH 7.4 adjusted with NaOH). The intracellular pipette solution contained 140 mM Cs-Aspartate, 8 mM MgCl_2_, and 10 mM HEPES (290–300 mOsm, pH 7.2 adjusted with CsOH) and either 1.2 mM EGTA or 10 mM BAPTA to enable or avoid SCDI.

### Ca^2+^ imaging

HEK-293 cells, Jurkat cells, or BV-2 cells were seeded onto 18 mm coverslips embedded in 4-well plates and loaded with 2 µM Fura-2 AM dissolved in a normal physiological salt solution (135 NaCl, 5 KCl, 1 MgCl_2_, 1.2 CaCl_2_, 10 HEPES, 5.5 glucose, and titrated pH 7.4 with NaOH) for 30 min at room temperature. The chamber was placed on the IX-73 inverted microscope platform (Olympus, Tokyo, Japan) attached to a complementary metal-oxide-semiconductor camera and a LED illuminator (pE-340fura, CoolLED Ltd., Andover, UK) and maintained at 37 °C using temperature controller (CU-501, Live Cell Instrument, Korea). The fluorophore was alternately excited at 340 and 380 nm while emission was recorded at 510 nm using MetaFluor 7.10.2 (Molecular Devices). At the end of each experiment, 10 µM ionomycin was added to induce a maximal ratio for comparison between control and treated cells. To measure ER Ca^2+^ and peri-lysosomal Ca^2+^, we used G-CEPIA1er and GCaMP6-TPC2, respectively, introduced by transfection 48 hours prior to the experiment, and fluorescence excitation at 488 nm and emission at 535 nm.

### FRET experiments

The FRET assay was performed by mRuby3-STIM1 and mClover3-STIM1 transiently expressed in HEK-293 cells using Lipofectamine 2000. Images were acquired on IX-73 inverted microscope (Olympus) with pE-340fura illuminator (CoolLED Ltd) at 37 °C. For mClover3, 488 nm excitation was used, with 500- to 600-nm light collected; for mRuby3, 559-nm excitation was used, with 570- to 670-nm light collected. For the FRET signal, 488-nm laser excitation was used, with 570- to 670-nm light collected. The FRET signal was calculated using MetaMorph software (Molecular Devices).

### TIRF experiments

TIRF imaging was performed on a Nikon Eclipse Ti microscope equipped with a Perfect Focus System (PFS III) using a 100× oil CFI Apochromat TIRF Objective (NA 1.49; Nikon Instruments Europe) and a ZET 561/10 excitation filter (Chroma Technology Corp., Bellows Falls, VT). HEK cells were transiently transfected with RFP-STIM1 or truncated (1-448) mutant STIM1 tagged with mCherry. The fluorescence was collected by an EMCCD camera cooled at −80 °C (iXon Ultra 897, Andor Technology Ltd) piloted with NIS-Elements Ar software V4.13 (Nikon). The PM was labeled with deep red CellMask (1:10 000 in CA for 10 min at RT) and used to adjust the TIRF angle. All experiments were performed at room temperature (22–25 °C).

### Phagocytosis

BV2 microglial cells were plated in 18 mm coverslips and pretreated with lipopolysaccharide (Sigma Aldrich) for 24 h. After application of TPC2-A1-N or GSK-7975A for 20 min, 50 μg of fluorogenic E. coli particles (pHrodo™ Green, P35366, Thermo Fischer) were added for 20 min at 37 °C. Fluorescence and phase contrast images were acquired on a Zeiss LSM 800 confocal microscope with ZEN2.3 software. To quantify the fluorescence intensity and cell area, ImageJ software from NIH was employed.

### Western blot

For total protein extraction, cells seeded on six-well plates were washed with ice-cold PBS and lysed with cold RIPA buffer containing phosphatase and protease inhibitor cocktail. The supernatants from lysates were loaded for SDS-PAGE and then transferred to polyvinylidene difluoride membrane (Merck Millipore, Billerica, MA). The membrane was blocked by 6 % skim milk for 1 hour at room temperature, followed by incubation with primary antibody at 4 °C overnight Antibodies for IP_3_R1 (#8568, Cell Signaling Technology, Danvers, MA) and STIM1 (#5668, Cell Signaling Technology) TPC2 (#119915, Abcam, Cambridge, UK), and β-actin (#20536-1-AP, Proteintech Inc., Rosemont, IL) were used. Horseradish peroxidase (HRP)-conjugated secondary antibody against either mouse or rabbit IgG was incubated for 1 hour at room temperature. The bands were visualized with a ChemiDocTM XRS+ imaging system (Bio-Rad) using enhanced chemiluminescence (ECL) solution (Luminata Forte, Millipore, Billerica, MA).

### Quantitative PCR

Total RNA was extracted from HEK-293 cells using Hybrid-R RNA kit. cDNA was synthesized from 0.5–1 g RNA with qPCR RT Mastermix according to the manufacturer’s protocol. cDNA was amplified using Accupower HotStart PCR premix (Bioneer, Daejeon, Korea) to detect the expression of genes of interest. For real-time PCR, each cDNA sample was mixed with SYBR Green PCR mastermix in a triplicate manner in a real-time PCR machine. Sequences of forward and reverse primers for TPCN2 are AAGGTGCGTTCCTATGGCAGTG and GCAGAAACGGAGACTGGTACTC, and for CALM1 are CCAACAGAAGCTGAATTGCAGGA and CAAAGACTCGGAATGCCTCACG. ΔΔCt method using Rn18s as an internal control was used to analyze relative expression.

### Knockdown experiments

HEK-293 cells were transfected with 100 nM small interfering RNA (siRNA) duplexes (Bioneer) from human TPCN2, or a negative control mixed with DharmaFECT siRNA transfection reagent. Sequences of 4 siRNAs targeting human TPCN2 are GGAAGCCUGUUUCUGAUGA, UGACAGAAGUGUGGUUAAA, GGUGGGACCUCUGCAUUGA, and GGUGGUCUACUACGUAUUU, and siRNA for siCALM1 is from (#17646-00-0005, Dharmacon, Lafayette, CO, USA). The transfection efficacy was assessed after 48 h by quantitative PCR.

### Plate reader for Ca^2+^ measurements

Intracellular Ca²⁺ measurements were performed using an FDSS µCell functional drug screening kinetic plate reader (Hamamatsu) in 384-well format. HEK293T cells were loaded at 2 × 10⁶ cells mL^−^¹ with 4 μM Cal-520 AM (AAT Bioquest) in Ca²⁺ assay (CA) buffer (140 mM NaCl, 5 mM KCl, 1 mM MgCl₂, 20 mM HEPES, 2 mM CaCl₂, 10 mM glucose, pH 7.4) for 45 min at room temperature in the dark. Cells were washed and resuspended in CA buffer at 1 × 10⁶ cells mL. Loaded cells (30,000 cells in 30 μL per well) were dispensed into 384-well plates (Corning CellBIND, #3770). EGTA (5 μL, 21 mM; final concentration 3 mM) was added and incubated for 2.5 min prior to fluorescence acquisition. Basal fluorescence under Ca²⁺-free conditions was then recorded for 30 s (excitation 485 nm, emission 520 nm; sampling interval 500 ms). Subsequently, 5 μL thapsigargin (8 μM; final concentration 1 μM) was added for 7 min to deplete intracellular Ca²⁺ stores, followed by addition of test compounds (TPC2-A1-N, TPCA1-P, and GSK-7975A; 5 μL, serial dilutions) for 5 min. Store-operated Ca²⁺ entry (SOCE) was then induced by addition of 5 μL CaCl₂ (40 mM), and the peak Ca²⁺ response was used for quantification. Compounds were tested either independently or in combination at equivalent molar ratio.

### Statistical analysis

All experiments were conducted with randomized samples and expressed as mean ± standard deviation (SD) or standard error of the mean (SEM). Using Prism software (Version 9.1.0.221, GP9-1988787-RLRM-FA1F1, GraphPad Software, San Diego, CA, USA), unpaired two-tailed Student’s *t* test or one-way analysis of variance (ANOVA) were performed. Post-hoc multiple comparison test after ANOVA were performed. A p value of less than 0.05 was considered statistically significant.

### Reporting summary

Further information on research design is available in the Nature Portfolio Reporting Summary linked to this article.

## Supplementary information

Supplementary Information

Reporting Summary

Transparent Peer Review file

## Source data

Source Data

## Data Availability

The data that support the findings of this study are presented in the main and Supplementary Figs., with number of experiments and statistical tests applied. Primary data are available from the corresponding authors upon reasonable request. Source data are provided with the paper. Source data are provided with this paper.
