# Supplementary material for: Two-pore channel-2 controls calmodulin-dependent STIM1 inactivation
Source: Nat Commun. 2026 Jul 1;17:8186. doi: 10.1038/s41467-026-75158-7 (PMC13462750; doi:10.1038/s41467-026-75158-7)
Supplement: Supplementary file 1 [file 41467_2026_75158_MOESM1_ESM.pdf]

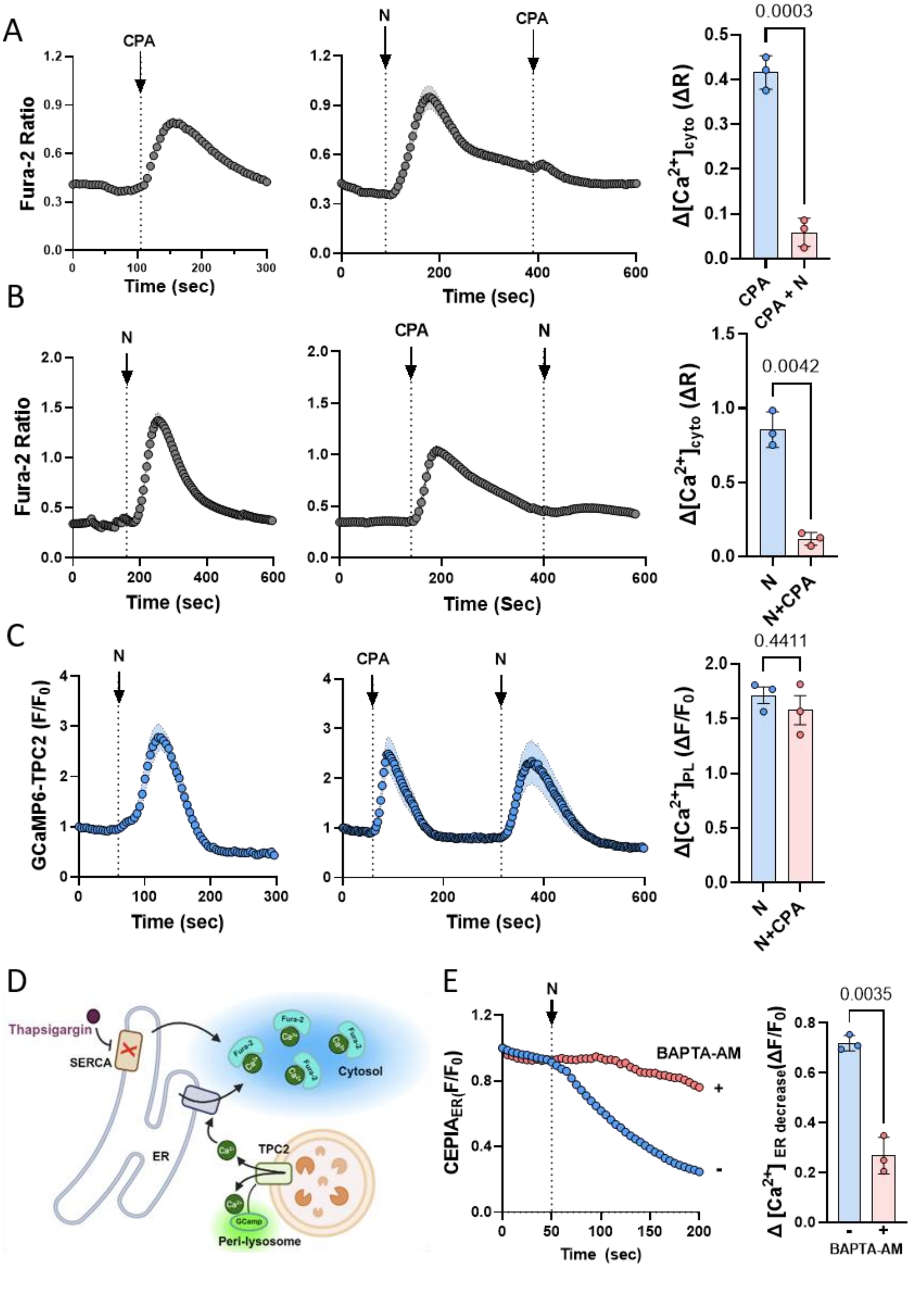

Supplementary Figure 1. TPC2-A1-N induces lysosomal Ca^{2+} release.

(A-B) Averaged Fura-2 ratio recordings (left panels) and quantification of $[Ca^{2+}]_{cyt}$ elevations (right) evoked by TPC2-A1-N (N, 20 μM) before ER stores depletion with CPA (10 μM) (A) and N after CPA (B) in HEK-293 cells.

(C) Averaged GCaMP6-TPC2 recordings (left panels) and quantification of peri-lysosomal $[Ca^{2+}]$ elevations (right) evoked by N before and after CPA application.

(D) Scheme showing the measurement of cytosol or peri-lysosome Ca^{2+} recordings, created in BioRender. Park, K. (2026) https://BioRender.com/ajh1atv.

(E) Effect of pretreatment with BAPTA-AM, a cell membrane permeant Ca^{2+} chelator, on CPA (red trace)- and TPC2-A1-N (blue trace)-mediated ER Ca^{2+} release. $CEPIA_{ER}$ recordings and quantification from cells.

Data are mean ±SD of N = 3 independent recordings from 257/205 cells for each group (A), 171/181 cells (B), 60/59 (C), and 109/ 114 cells (E), two-tailed unpaired t test with Welch's correction.

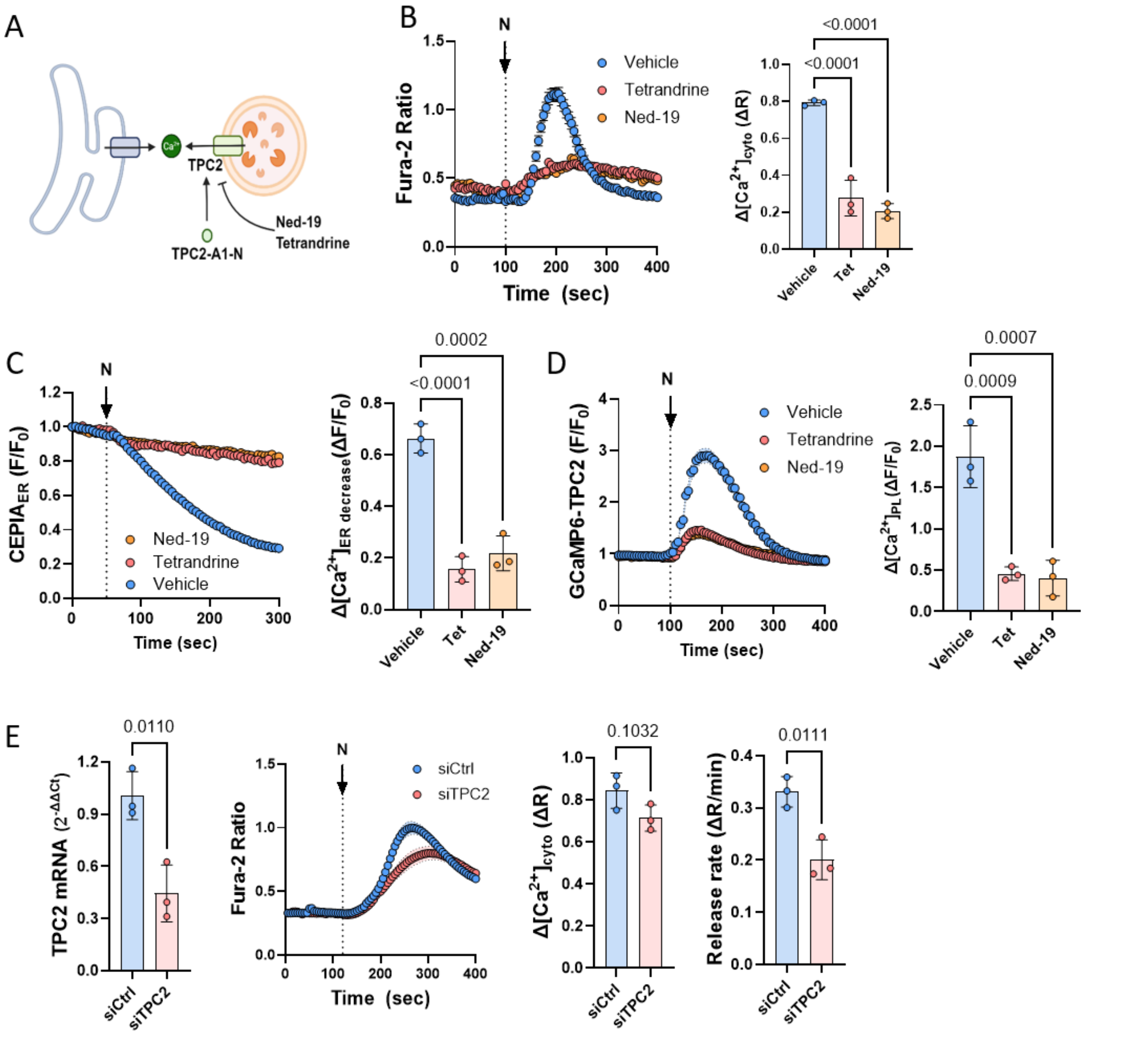

Supplementary Figure 2. TPC2-A1-N-induced lysosomal and ER Ca^{2+} release is blocked by TPC2 inhibitors.

(A) Scheme showing the intracellular targets of the agonist and inhibitors applied, created in BioRender. Park, K. (2026) https://BioRender.com/0sncauz.

(B-D) Averaged Fura-2 ratio (B), $CEPIA_{ER}$ (C), and GCaMP6-TPC2 (D) recordings and quantification of $[Ca^{2+}]_{cyt}$, $[Ca^{2+}]_{ER}$, and $[Ca^{2+}]_{peri\text{-}lyso}$ changes evoked by N in cells treated or not with Ned-19 (5 µM, orange traces) or Tetrandrine (3 µM, red traces) to inhibit TPC2 channels.

(E) TPC2 mRNA levels by RT-qPCR (left), averaged Fura-2 ratio recordings (middle) and quantification of $[Ca^{2+}]_{cyt}$ elevations (right) evoked by N in cells transfected with scrambled and TPC2 targeting si-RNAs. Data are mean ±SD of N = 3 independent recordings from 171/174 cells for each group (B), 106/102 cells (C), 61/55 cells (D), and 136/109 cells (E), two-tailed unpaired t test with Welch's correction.

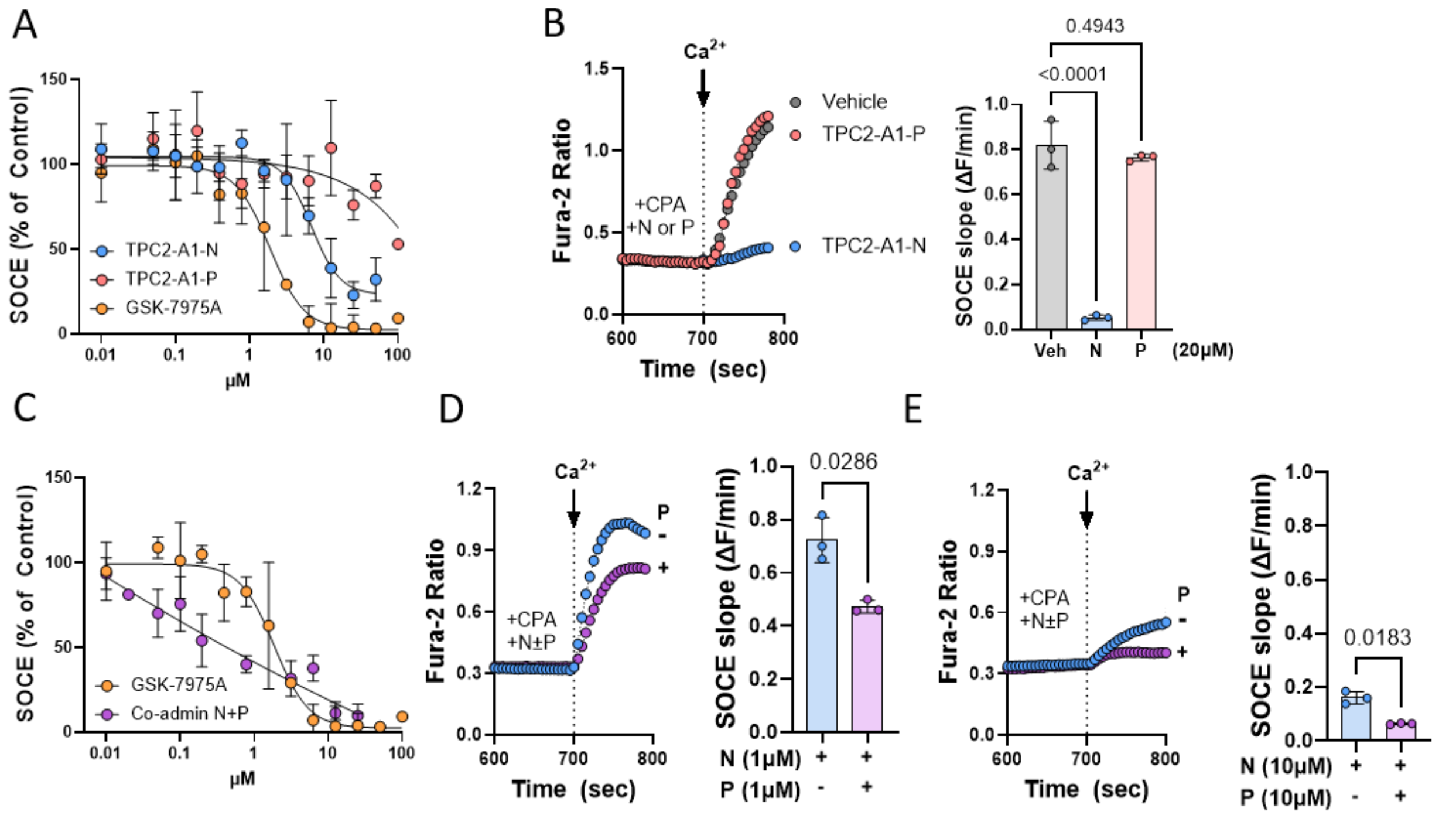

Supplementary Figure 3. Concentration–response relationships for TPC2 activators.

(A) Concentration–response inhibition curves of TPC2-A1-N (N) and TPCA1-P (P) on store-operated Ca^{2+} entry (SOCE) in HEK293T cells. GSK-7975A was used as a reference Orai1 channel inhibitor.

(B) Averaged Fura-2 ratio recordings (left) and quantification of Ca^{2+} entry rates (right) in cells treated with CPA followed by 20 μM N or P for 5 min before Ca^{2+} readmission.

(C) Concentration–response inhibition curves of the combination of N and P on SOCE in HEK293T cells, with GSK-7975A as a reference inhibitor.

(D-E) Averaged Fura-2 ratio recordings in cells treated with CPA followed by the combination of 1 μM N + 1 μM P or 10 μM N + 10 μM P for 5 min before Ca^{2+} readmission.

Data are mean ±SD of N = 4 independent biological replicates, each measured in technical triplicate (A,C), and N = 3 independent recordings from 188/198/209 cells for each group in the bar graph (B), ordinary one-way ANOVA with Dunnett's multiple comparisons test, and 205/235 cells (D) and 242/215 cells (E), two-tailed unpaired t test with Welch's correction.

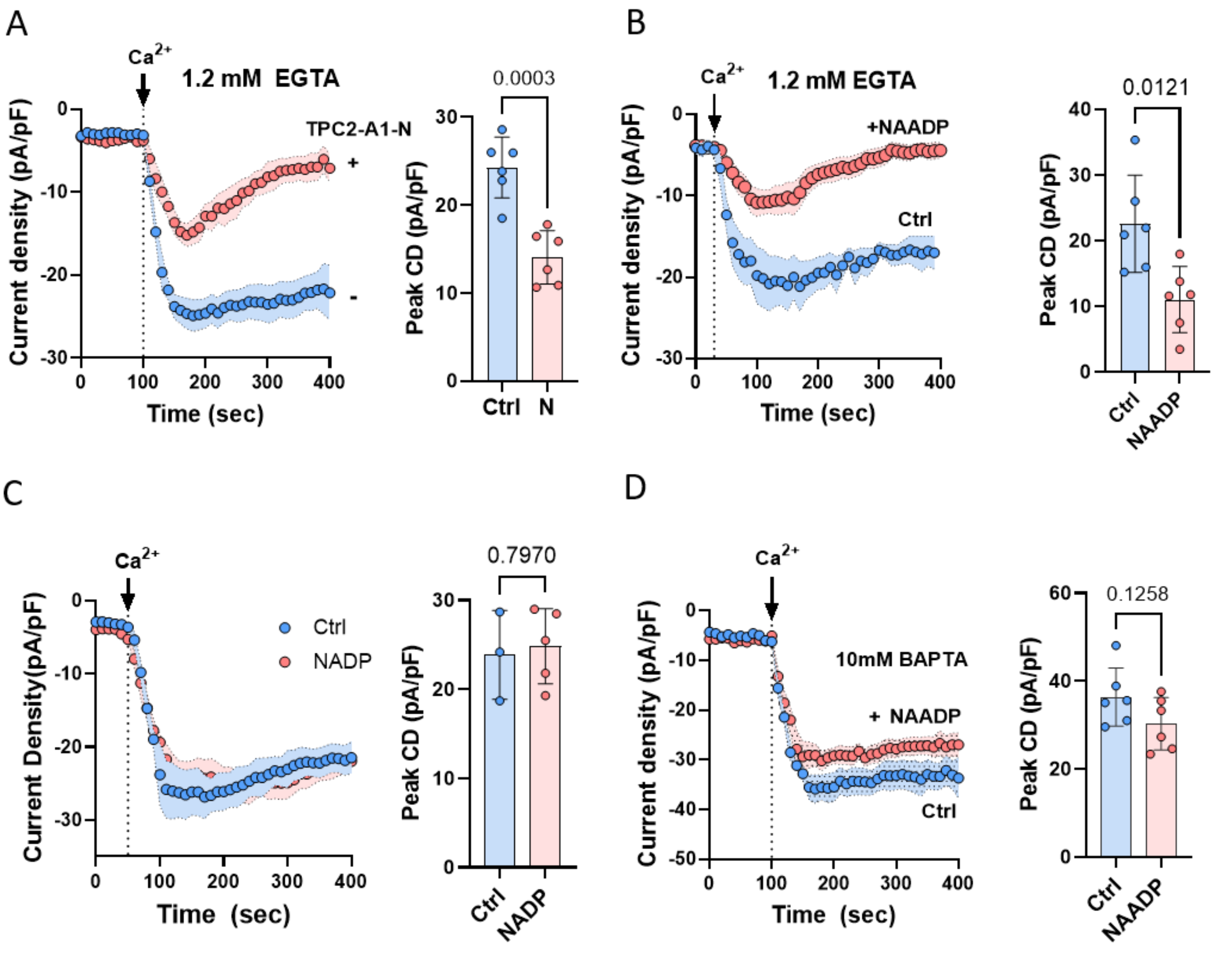

Supplementary Figure 4. TPC2 activation enhances the SCDI of STIM1-gated Orai1 channels

(A-C) Time-course (left) and peak current densities (right) of the averaged whole-cell currents shown in Fig. 4B-D.

(D) Time-course (left) and peak current densities (right) of the averaged whole-cell currents with pipette solutions containing or not NADP.

Data are mean ±SEM of N = 6 recordings (A-C) or 3/5 recordings (D), two-tailed unpaired t test with Welch's correction.

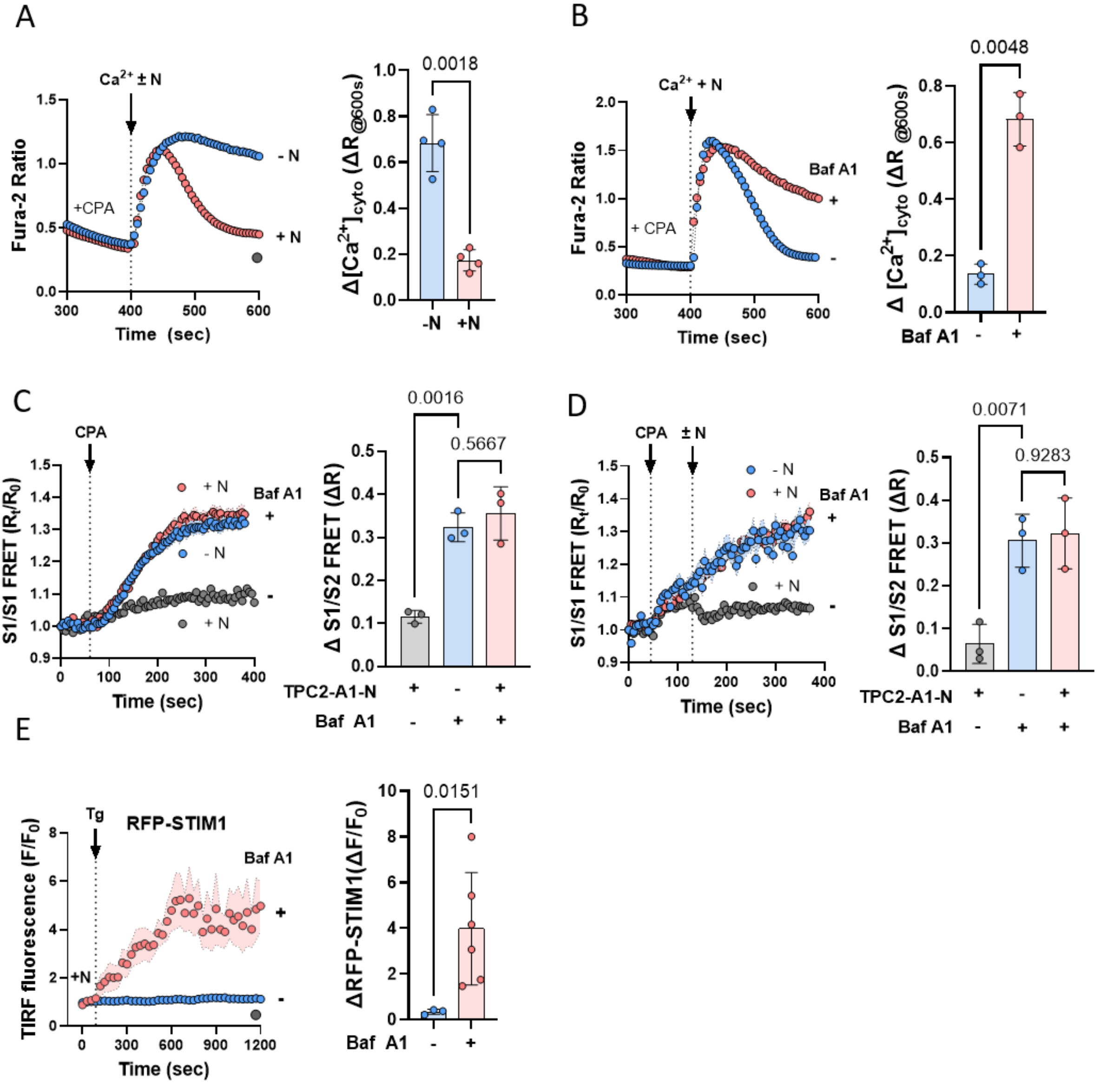

Supplementary Figure 5. Lysosomal Ca^{2+}-dependent SOCE inhibition by TPC2-A1-N.

(A-B) Averaged Fura-2 ratio recordings (left) and quantification of SOCE decay rates (right) in co-treatment of CPA with (red trace) or without (blue trace) 20 µM TPC2-A1-N (A), and pretreatment (red trace) or not (blue trace) with bafilomycin A1 (Baf A1) (B).

(C-D) Changes in mRuby3-STIM1/mClover3-STIM1 FRET fluorescence evoked by CPA (10 µM) in cells pretreated with Baf A1 and N (red trace), Baf A1 only (blue trace), or N only (grey traces) (C). Acute effects of N applied 100 sec after CPA addition on FRET fluorescence (D)

(E) Changes in RFP-STIM1 TIRF fluorescence evoked by 1 µM Tg in cells pretreated with N and Baf A1 (red trace) or N only (blue trace).

Data are mean ±SD of N =3 independent recordings from 141/103 cells for each group in the bar graph (the same applies below) (A), 152/171 cells (B), 40/30/32 cells (C), 39/22/24 cells (D), and N = 2/6 cells (E), two-tailed unpaired t test with Welch's correction (A,B,E) and ordinary one-way ANOVA with Dunnett's multiple comparisons test (C,D).

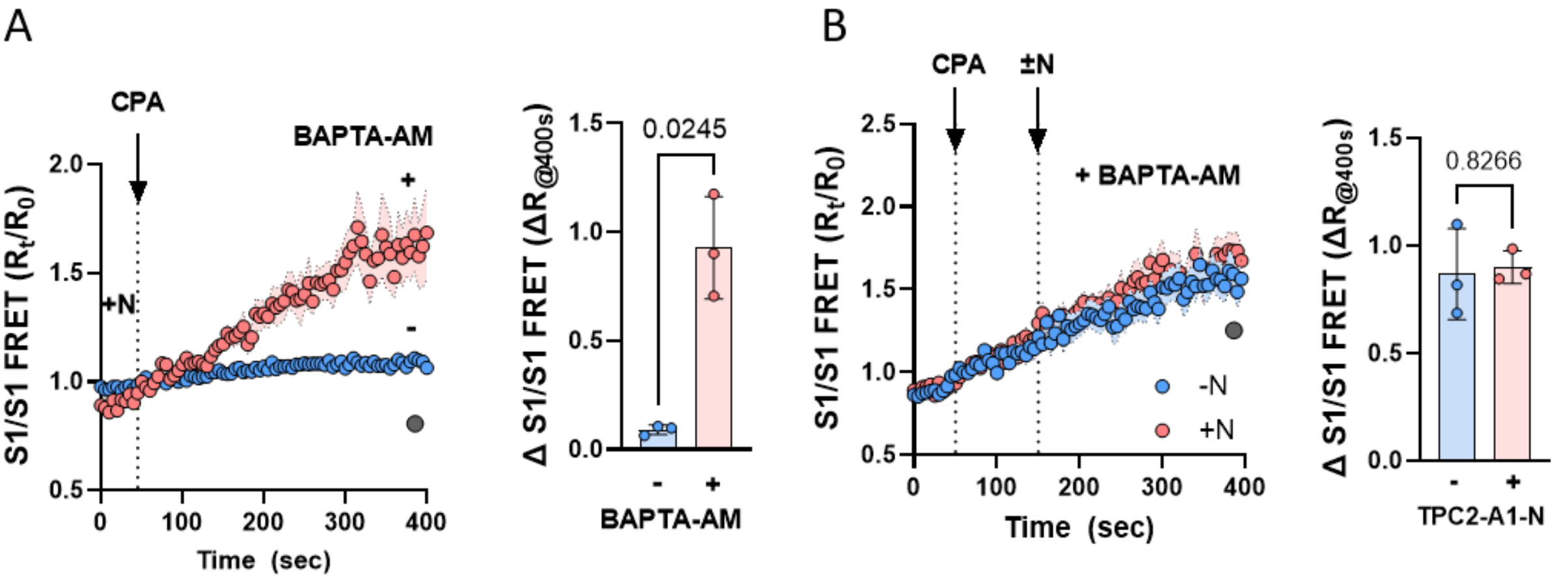

Supplementary Figure 6. Calmodulin and the SOAR domain of STIM1 mediate TPC2-dependent STIM1 inactivation

(A-B) Changes in mRuby3-STIM1/mClover3-STIM1 FRET fluorescence evoked by CPA (10 µM) in cells pretreated with N (20 µM) exposed or not to 40 µM BAPTA-AM for 5 min (A) and applied N 100 sec after CPA (B). Data are mean ±SD of N = 3 independent recordings from 60/49 cells for each group in the bar graph (D) and 20/19 cells, two-tailed unpaired t test with Welch's correction.
