# Supplementary material for: Two-pore channel-2 controls calmodulin-dependent STIM1 inactivation
Source: Nat Commun. 2026 Jul 1;17:8186. doi: 10.1038/s41467-026-75158-7 (PMC13462750; doi:10.1038/s41467-026-75158-7)
Supplement: Supplementary file 3 [file 41467_2026_75158_MOESM3_ESM.pdf]

nature portfolio

Peer Review File

Two-pore channel-2 controls calmodulin-dependent STIM1 inactivation

Corresponding Author: Professor Nicolas Demaurex

This file contains all reviewer reports in order by version, followed by all author rebuttals in order by version.

Version 0:

Reviewer comments:

Reviewer #1

(Remarks to the Author)
This manuscript investigates the role of two-pore channel 2 on STIM1 activation and SOCE. The authors show that TPC2 activation, using the agonists NAADP and TPC2-A1-N, inhibits SOCE in different cell lines by enhancing the slow Ca2+-dependent inactivation of Orai1 channels. TPC2 activation mobilizes ER Ca2+ stores independently of IP3R by a mechanism involving RyRs. In addition, TPC2 activation impairs STIM1 oligomerization (or disassembles STIM1 clusters) and prevents RFP-STIM1 recruitment to the TIRF plane upon ER depletion using thapsigargin, which attenuates both calcium and manganese influx. These effects persisted in STIM1 mutants truncated after the SOAR and were prevented by pharmacological inhibition of calmodulin. The issue investigated is interesting and timely and the manuscript is nicely written and organized. There are some points that need attention.
Major concerns:
1. In Fig 1B the authors show ER calcium store discharge upon activation of TPC2 receptor in the absence of detectable SOCE, while TG induces similar cytosolic calcium elevation and significant SOCE. First, the comparison of calcium release by Tg or using the TPC2 agonist is not appropriate as the elevation in cytosolic calcium concentration by TPC2 agonists is due to calcium release from the lysosomes as well as from the ER while TG releases calcium predominantly from the ER and the rate of calcium discharge from the ER is not comparable as observed in Fig 1C. Slow discharge of the ER might induce a slow SOCE, potentially involving STIM2, that is outside the detection range of Fura-2. To address this issue the authors should express Orai1-fused with a GECI with an appropriate kd for cytosolic calcium.
2. Concerning Fig. S1 what is the explanation for the lack of response of the TPC2 agonist when applied after CPA in Fig S1B, which contradicts the results using GCaMP6-TPC2 (Fig S1C).
3. Fig. 1D should show the protein expression of the TPC2 mutants as well as native TPC2 in the same cells (e.g., via Western Blot).
4. HEK-293 cells express very low levels of endogenous RyRs. To strengthen their hypothesis the authors should demonstrate calcium mobilization by low concentrations of ryanodine. In addition, the role of ryanodine in TPC2-induced ER calcium mobilization should be further tested using a high ryanodine concentration. The effect of TPC2-induced calcium elevation on endogenous ryanodine receptors should be further tested using the fast intracellular calcium chelator BAPTA and assessing ER calcium concentration directly with ER-targeted sensors like CEPIA.
5. The results show that treatment with TPC2-A1-N attenuates STIM1 oligomerization and TIRF. Is this effect prevented by treatment with bafilomycin A1? These experiments could clarify whether lysosomal calcium release triggers this process.
6. To strengthen the hypothesis regarding the role of CaM in TPC2-mediated inhibition of STIM1 oligomerization, a CaM knockdown should be performed in addition to the use of pharmacological inhibitors.
Minor:
1. Figures 3C and 3D are swapped in the Results section.
2. Fig. 3E shows that TPC2-A1-N induces calcium elevation, as a result of calcium release from the lysosomes, while this is expected, do the authors have an explanation for the lack of response in Fig 3A?

Reviewer #2

(Remarks to the Author)
This is a very interesting paper showing how activation of lysosomal TPC2 channels inhibits store-operated Ca2+ entry. There is (too?) much interest in mechanisms underpinning SOCE. And there is a rapidly growing interest in lysosomal

calcium signaling. Connecting the two is exciting and impactful. This paper is well presented and the data convincing providing mechanism and some function. I have several comments that the authors might consider for improving what is already a fine study.

Major.

The authors relay heavily on TPC2-A1-N to draw their conclusions and have done many controls to probe specificity. I suggest testing TPC2-A1-P. This compound is less effective at raising bulk calcium levels that TPC2-A1-N so it would be very interesting to see what it does to SOCE. Despite such segregated action of the two agonists, they act synergistically to boost calcium signaling through TPC2. Again, it would be interesting to see what the 'combo' does.

Figure 2. The authors show convincingly that TPC2-A1-N-evoked calcium signals are IP3R independent and instead blocked by dantrolene, an RyR inhibitor. This is a little confusing as previous work showed that IP3R KO delayed bulk N-evoked calcium signals (ref 14 in the paper). Also, the reviewer was under the impression that HEK cells do not express functional RyRs. I suggest removing these data until this point is clarified. The bafilomycin data in this figure is very important showing specificity of the N compound. So, I would move those data to Figure 1.

Minor.

Figure 1D. This shows nicely that the TPC2 pore mutants act as dominant negatives on bulk calcium. But it is less clear what overexpression of TPC2 does here as there is no appropriate control (inert over-expressed protein/mock transfection). Previous work suggested that overexpressing TPC2 increases the rate of Ca2+ release by N not the amplitude (ref 14).

Fig. 1 Legend. It is stated 'Data are mean ±SD of N = 3-4 independent recordings from 130/101 cells (B), 171/174, 106/102, and 61/55 cells (C-E), 189/196, 121/100, and 58/55 cells (F-H), two-tailed unpaired t test with Welch's correction' What does '130/101' mean? Number of cells from 2 experiments? But it is stated that data are from 3-4 experiments. If these experiments are truly independent then perhaps s.e.m should be used. Please clarify, maybe as a section in the methods.

Figure 2A. The author have succeeded in picking up endogenous TPC2 by Western blotting (something the reviewer' s lab struggles with!). It would be nice to see what siRNA (Fig. S1) does here if the authors decide on retaining these data (see major point 2).

Fig.3F. Please add a control for TPC2 expression here so we can see the extent of reversal by the TPC2 mutants.

Fig. S3A. The finding that bafilomycin reverses N block is important. Please bring this into the main Figure perhaps by replacing the dantrolene data, given my concerns above about ryanodine receptors.

Figure 4. It would be nice to see some negative controls here on CRAC currents. Can the authors test the effects of TPC2-A1-P (major point 1) and NADP?

Figure 5. As the authors quite rightly state, TPC2 activation 'remarkably' inhibits on going SOCE. it would be prudent to test specificity of the compound here using some of the strategies applied earlier. This is because it is difficult to explain at present how a presumed local calcium signal can have a functional effect in the face of global calcium influx.

Figure 7. Here, Jurkat cells are pretreated with N and this is shown to block physiological Ca2+ responses to T cell activation. Can we please see what N does acutely as supp data? I ask because there have been unpublished reports at scientific meetings showing that N induces robust non-specific Ca2+ entry in these cells.

Discussion: The authors should discuss this paper as it is very relevant to some of the effects reported here: https://pubmed.ncbi.nlm.nih.gov/33328326/

Really minor!

Abstract: It is stated 'The TPC2 agonists NAADP and TPC2-A1-N...' I suggest using 'activators' to make a subtle distinction between the two given that NAADP acts indirectly through NAADP binding proteins.

Introduction: Please reference this sentence: 'TPC2 is a dual-gated ion channel of late endosomes and lysosomes comprising two homologous domains that dimerize to form a functional "quasitetrameric" channel whose selectivity is agonist-dependent, switching between Na+ and Ca2+ based on the stimulus.'

Introduction: Consider deleting the following sentence as the authors of the referenced work find no role for TPC2 in T cells: 'In T-cells, JPT2 coprecipitates with type I ryanodine receptor (RyR) on the ER and with T cell receptors on the cell membrane and was proposed to coordinate the NAADP-mediated activation of lysosomal TPC2 and ER RyR channels that generate Ca2+ microdomains during the first seconds of T cell activation 28, 31. TPC2-RyR coupling amplifies NAADP-mediated Ca2+ signals in T-cells..'

Introduction: Please fix:'The later inactivation constrain..'.

Results: Please fix repetition in: 'Furthermore, BAPTA-AM prevented the acute inhibition of STIM1 oligomerization evoked

by TPC2-A1-N observed in Fig. 5C abrogated in the presence of BAPTA-AM (Fig. 6E).'

Discussion: Fix: ' PI(3,5)P3'

Version 1:

Reviewer comments:

Reviewer #1

(Remarks to the Author)
The authors have adequately addressed the previous concerns and improved/clarified their manuscript.

Reviewer #2

(Remarks to the Author)
I am happy with the authors response. Manuscript is now even stronger.
I don't want to see this again (for review purposes) but the authors should consider briefly discussing the inhibitory effect of TPC2-A1-N pretreatment on bead-evoked Ca2+ signals in T cells in calcium-free medium (Fig. 6). This implies that lysosomes regulate Ca2+ release as well as entry.
Also see https://pubmed.ncbi.nlm.nih.gov/33484198/ regarding possible TRPML1/STIM1 interaction.

Open Access This Peer Review File is licensed under a Creative Commons Attribution 4.0 International License, which permits use, sharing, adaptation, distribution and reproduction in any medium or format, as long as you give appropriate credit to the original author(s) and the source, provide a link to the Creative Commons license, and indicate if changes were made.
In cases where reviewers are anonymous, credit should be given to 'Anonymous Referee' and the source.
The images or other third party material in this Peer Review File are included in the article's Creative Commons license, unless indicated otherwise in a credit line to the material. If material is not included in the article's Creative Commons license and your intended use is not permitted by statutory regulation or exceeds the permitted use, you will need to obtain permission directly from the copyright holder.
To view a copy of this license, visit https://creativecommons.org/licenses/by/4.0/

Response to Reviewers Comments

We thank the Editors for the professional handling of our manuscript and the Reviewers for their insightful and constructive suggestions. Below is a point-by-point response to the critiques raised. For each comment the input of the Reviewer is listed in *italics* followed by our response in blue plain letters. In the interest of conciseness, only critical comments that require a response are listed.

Reviewer #1 (Remarks to the Author)

Major concerns:

1. In Fig 1B the authors show ER calcium store discharge upon activation of TPC2 receptor in the absence of detectable SOCE, while TG induces similar cytosolic calcium elevation and significant SOCE. First, the comparison of calcium release by Tg or using the TPC2 agonist is not appropriate as the elevation in cytosolic calcium concentration by TPC2 agonists is due to calcium release from the lysosomes as well as from the ER while TG releases calcium predominantly from the ER and the rate of calcium discharge from the ER is not comparable as observed in Fig 1C. Slow discharge of the ER might induce a slow SOCE, potentially involving STIM2, that is outside the detection range of Fura-2. To address this issue the authors should express Orai1-fused with a GECI with an appropriate kd for cytosolic calcium.

[Answer] We agree that a direct comparison of Ca^{2+} release responses is misleading as TPC2 agonists induce a slower ER Ca^{2+} depletion and additionally mobilize Ca^{2+} from lysosomes. The point of the figure was to illustrate graphically that TPC2 agonists efficiently deplete ER stores but fail to activate SOCE. To exclude contribution of STIM2-mediated SOCE we have performed the suggested experiments and recorded subplasmalemmal Ca^{2+} responses with Orai1-GCaMP6f. The new data (Fig. 1C) show that TPC2 agonists do not elicit detectable Ca^{2+} fluxes across the expressed Orai1 channel, while SERCA inhibition with CPA induces a very large response.

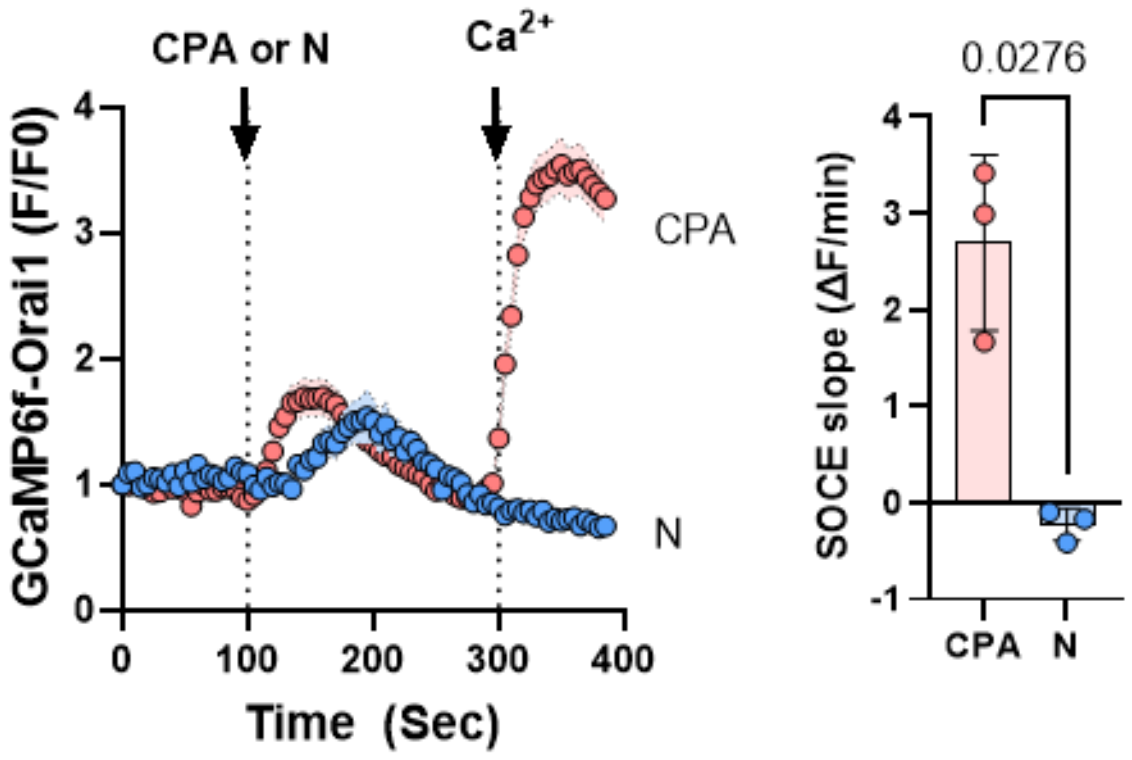

Figure 1C in the revised manuscript

Concerning Fig. S1 what is the explanation for the lack of response of the TPC2 agonist when applied after CPA in Fig S1B, which contradicts the results using GCaMP6-TPC2 (Fig S1C).

[Answer] As discussed in point 1 above, TPC2 agonists mobilize Ca^{2+} from both the ER and lysosomes. Our observation that TPC2 agonists and SERCA inhibitors evoke identical cytosolic Ca^{2+} elevations indicates that the responses mostly reflect ER Ca^{2+} release,

consistent with the ER being a larger Ca^{2+} store than lysosomes. The small fura-2 response observed when N is added after CPA thus reflects the smaller size of the lysosomal store. When ER Ca^{2+} stores are empty, TPC2 agonists only generate local peri-lysosomal Ca^{2+} elevations, which are readily detected by GCaMP6-TPC2 but barely detected with fura-2.

3. Fig. 1D should show the protein expression of the TPC2 mutants as well as native TPC2 in the same cells (e.g., via Western Blot).

[Answer] Protein expression of both WT and mutant TPC2 was confirmed by Western blot analysis, revealing similar immunoreactive band patterns (Figure R1). Antibody specificity for TPC2 was validated in cells in which TPC2 expression had been silenced.

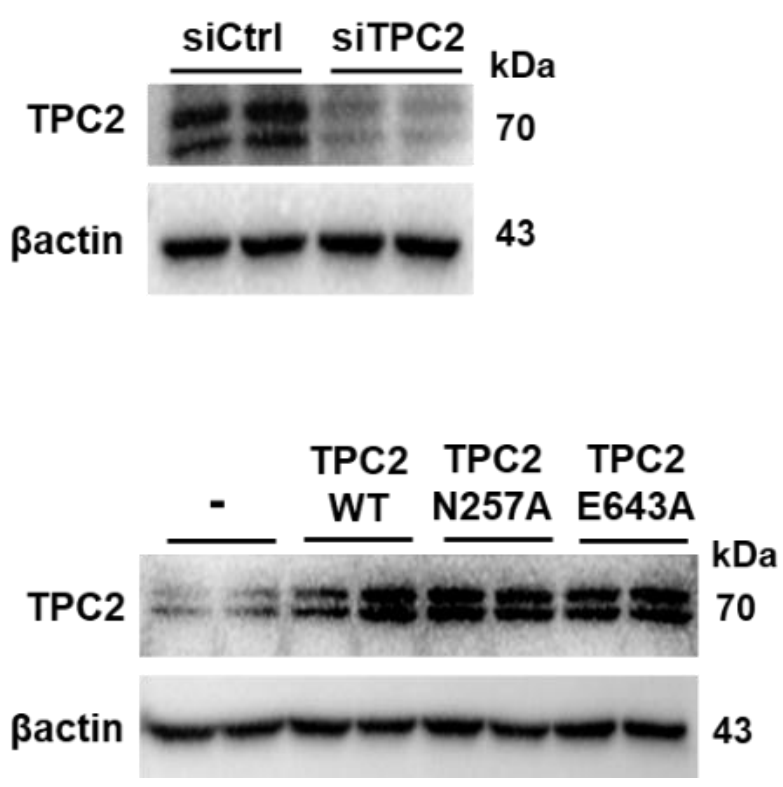

Figure R1. Representative Western blot showing TPC2 expression in HEK cells transfected with wild-type or mutant TPC2 using anti-TPC2 antibody.

4. HEK-293 cells express very low levels of endogenous RyRs. To strengthen their hypothesis the authors should demonstrate calcium mobilization by low concentrations of ryanodine. In addition, the role of ryanodine in TPC2-induced ER calcium mobilization should be further tested using a high ryanodine concentration. The effect of TPC2-induced calcium elevation on endogenous ryanodine receptors should be further tested using the fast intracellular calcium chelator BAPTA and assessing ER calcium concentration directly with ER-targeted sensors like CEPIA.

[Answer} We now show with ER-targeted CEPIA that BAPTA-AM prevents ER Ca^{2+} release evoked by TPC2-A1-N without impacting CPA-induced ER Ca^{2+} depletion (Fig. S1E). This establishes that the TPC2-induced ER Ca^{2+} mobilization reflects Ca^{2+}-induced Ca^{2+} release. To comply with the request of Reviewer #2 we have removed the dantrolene data and plan to clarify the relative contribution of RyR to TPC2-mediated ER Ca^{2+} release in a subsequent study.

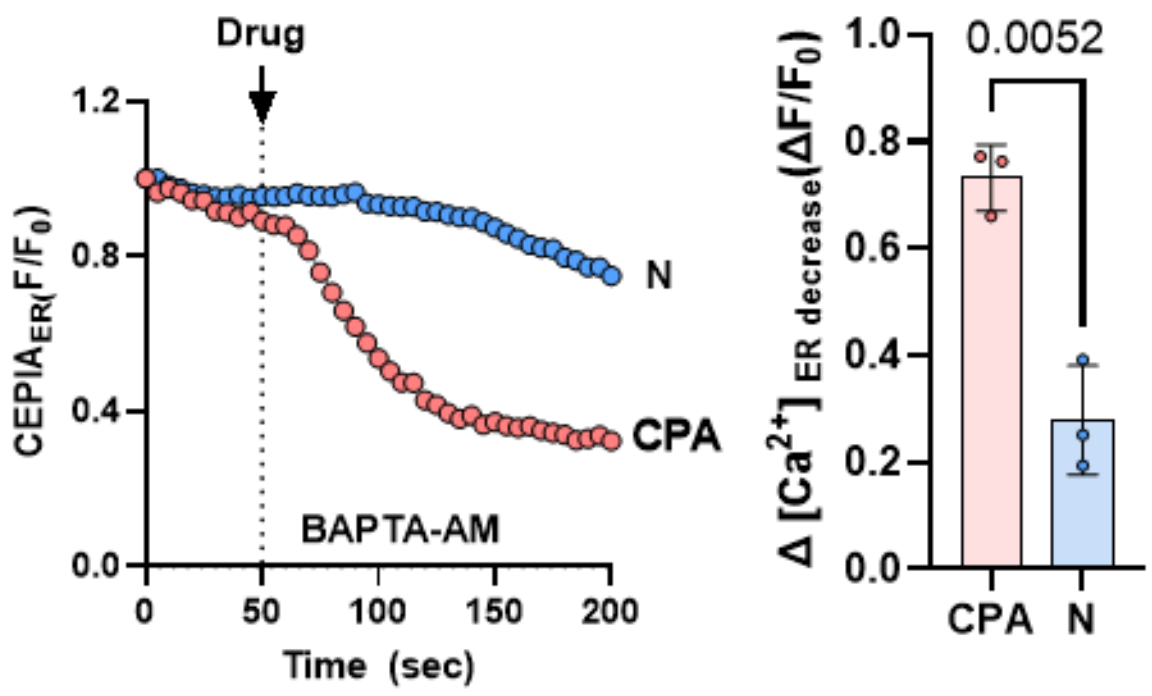

Figure S1E in the revised manuscript

5. The results show that treatment with TPC2-A1-N attenuates STIM1 oligomerization and TIRF. Is this effect prevented by treatment with bafilomycin A1? These experiments could clarify whether lysosomal calcium release triggers this process.

[Answer] We appreciate the reviewer's constructive comments. We now show the effect of bafilomycin A1 on STIM1 oligomerization and PM translocation. The new data (Fig. S4) confirm the involvement of lysosomes in mediating the TPC2-dependent dissociation of STIM1 and its retrieval from the PM.

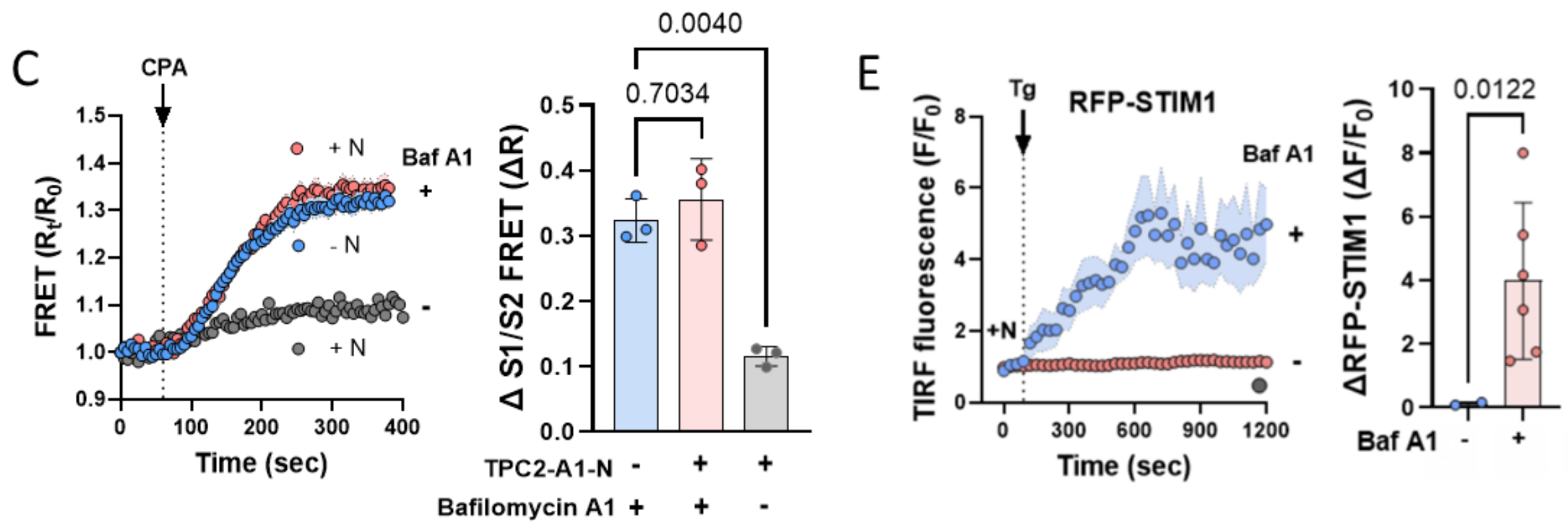

Figure S4 in the revised manuscript

6. To strengthen the hypothesis regarding the role of CaM in TPC2-mediated inhibition of STIM1 oligomerization, a CaM knockdown should be performed in addition to the use of pharmacological inhibitors.

[Answer] We now show that CALM1 silencing relieves the TPC2-mediated inhibition of SOCE (Fig. 5D). This confirms the implication of CaM in mediating the inhibition. We thank the reviewer for this suggestion.

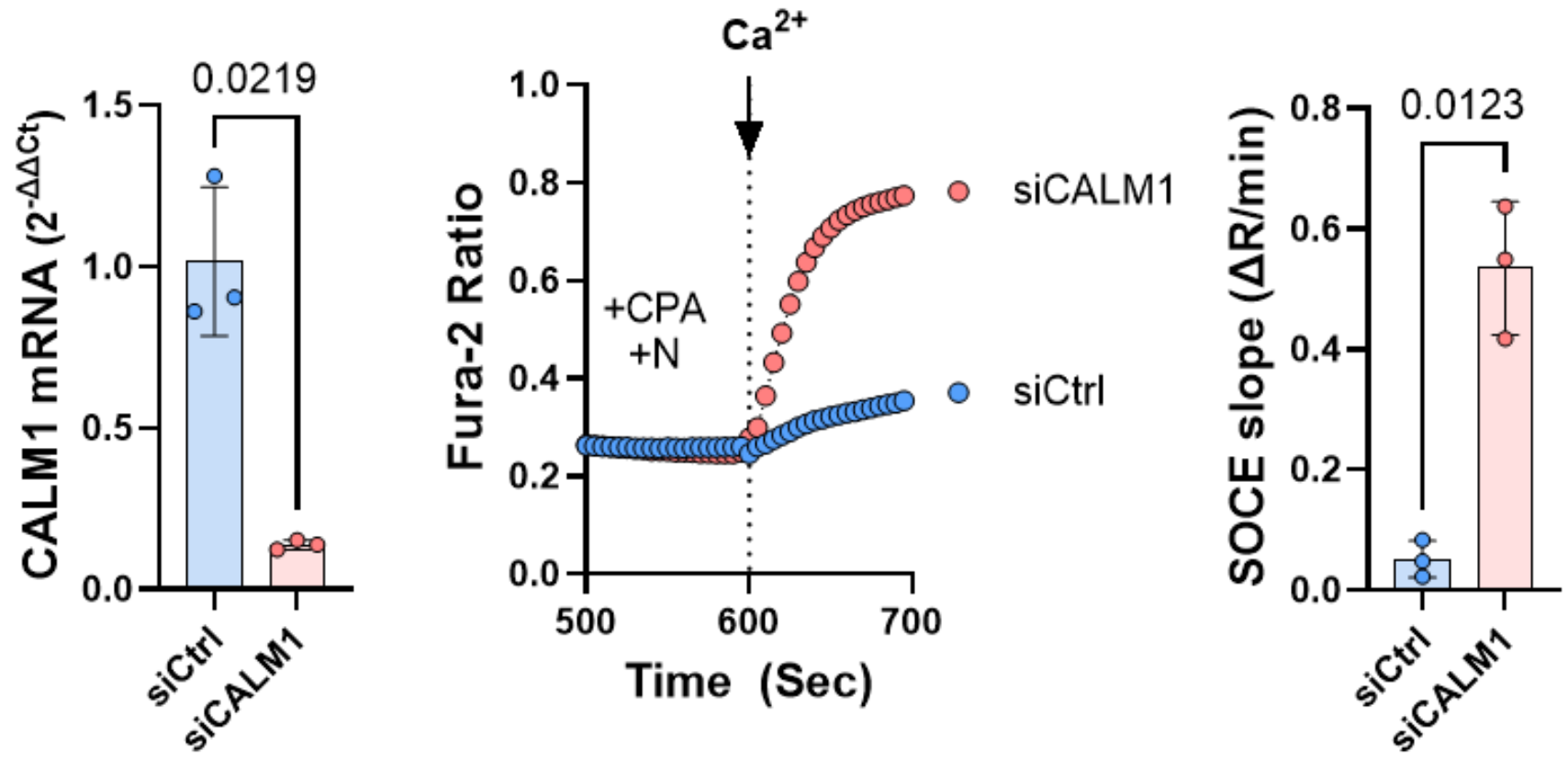

Figure 5D in the revised manuscript

Minor:

1. Figures 3C and 3D are swapped in the Results section. [Answer] Fixed

2. Fig. 3E shows that TPC2-A1-N induces calcium elevation, as a result of calcium release from the lysosomes, while this is expected, do the authors have an explanation for the lack of response in Fig 3A?

[Answer] As discussed in point 2 above, TPC2-A1-N evokes small cytosolic Ca^{2+} elevations when added after CPA because the larger ER store is depleted, leaving only the lysosomal contribution. A response of ~0.1 fura-2 ratio amplitude is visible in both Fig 3A and 3E (now Fig. 2A and 2E), but more apparent in Fig 3E with a two-fold reduction in the y-axis scale range. Residual responses of similar amplitude were observed in all cell types tested (Fig. R2 below).

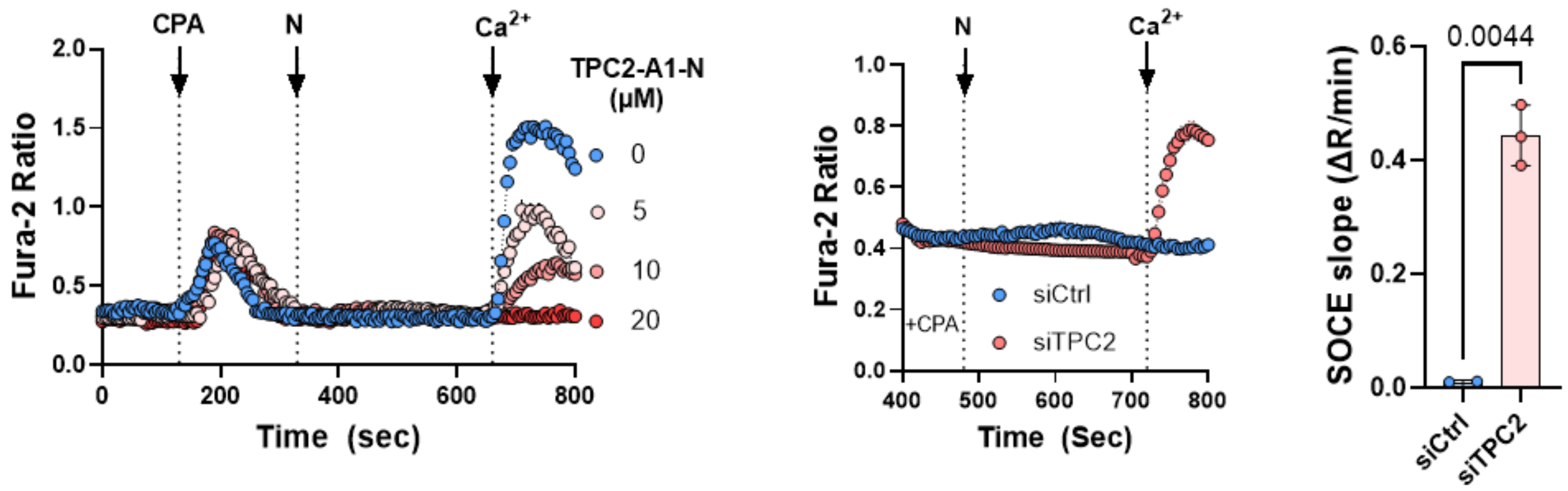

Figure 2A and 2E in the revised manuscript

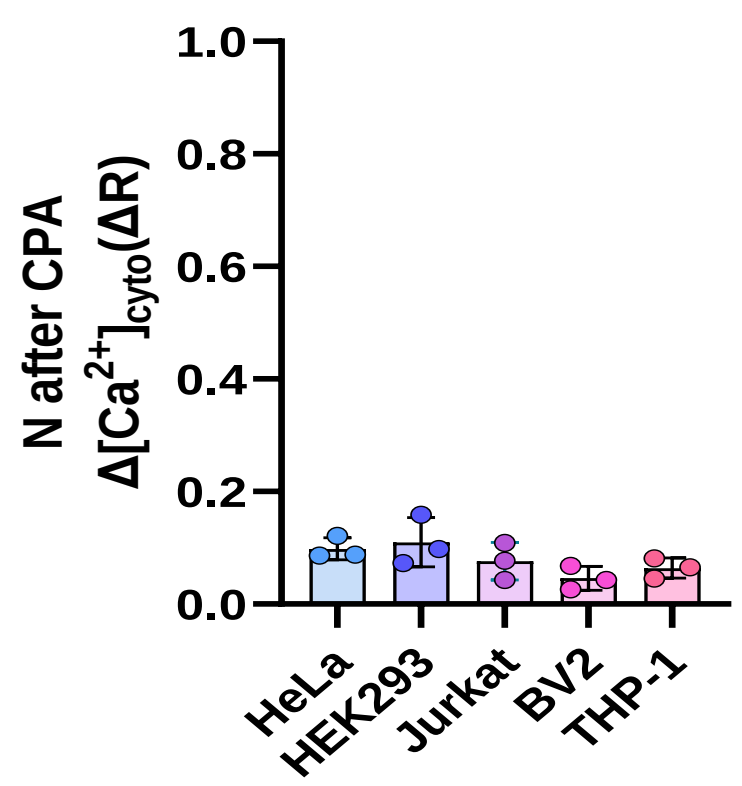

Figure R2. Amplitude of $[Ca^{2+}]_{cyt}$ elevations evoked by TPC2-A1-N in 5 different cell lines pre-treated with CPA to deplete ER Ca^{2+} stores. N= 3 independent recordings each.

Reviewer #2 (Remarks to the Author):

Major.

The authors relay heavily on TPC2-A1-N to draw their conclusions and have done many controls to probe specificity. I suggest testing TPC2-A1-P. This compound is less effective at raising bulk calcium levels that TPC2-A1-N so it would be very interesting to see what it does to SOCE. Despite such segregated action of the two agonists, they act synergistically to boost calcium signaling through TPC2. Again, it would be interesting to see what the 'combo' does.

[Answer] Thank you for your suggestion. We have obtained dose-responses for both compounds using a Hamamatsu microcell plate reader. A1-N was consistently more potent than A1-P in inhibiting SOCE and both were less potent than the reference Orai1 channel inhibitor GSK-7975A (Fig. S3A-B). A higher inhibition was observed with both compounds combined at identical concentrations (Fig. S3C-E).

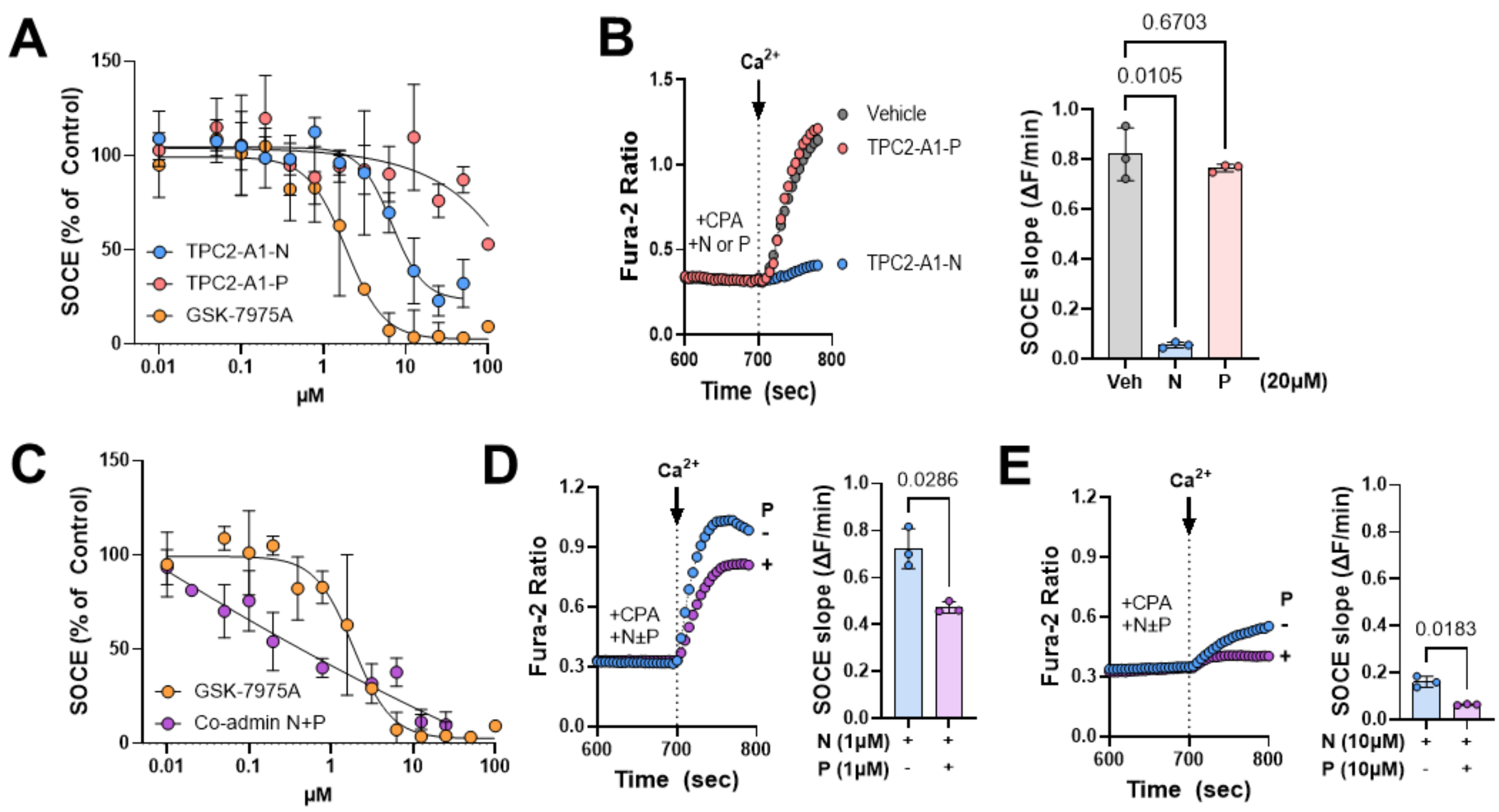

Figure S3 in the revised manuscript

Figure 2. The authors show convincingly that TPC2-A1-N-evoked calcium signals are IP3R independent and instead blocked by dantrolene, an RyR inhibitor. This is a little confusing as previous work showed that IP3R KO delayed bulk N-evoked calcium signals (ref 14 in the paper). Also, the reviewer was under the impression that HEK cells do not express functional RyRs. I suggest removing these data until this point is clarified. The bafilomycin data in this figure is very important showing specificity of the N compound. So, I would move those data to Figure 1.

[Answer] We were indeed surprised to observe rapid ER Ca^{2+} release in IP_3R KO cells as these cells, provided to us by David Yule, were previously reported to exhibit delayed Ca^{2+} elevations when exposed to TPC2-A1-N. The faster response and high dantrolene sensitivity that we observe might reflect an adaptation of these IP_3R-deficient cells to our culture

conditions. To comply with the reviewer's request, we have removed these data and plan to clarify the relative contribution of RyR and IP_3R to the ER Ca^{2+} release evoked by TPC2-A1-N in a subsequent study. As suggested, we have moved the bafilomycin data to Figure 1. We now also show that bafilomycin A1 completely relieves the acute and chronic effects of A1-N on SOCE as well as on STIM1 oligomerization and PM translocation (Fig. 4E-F and S5B-E).

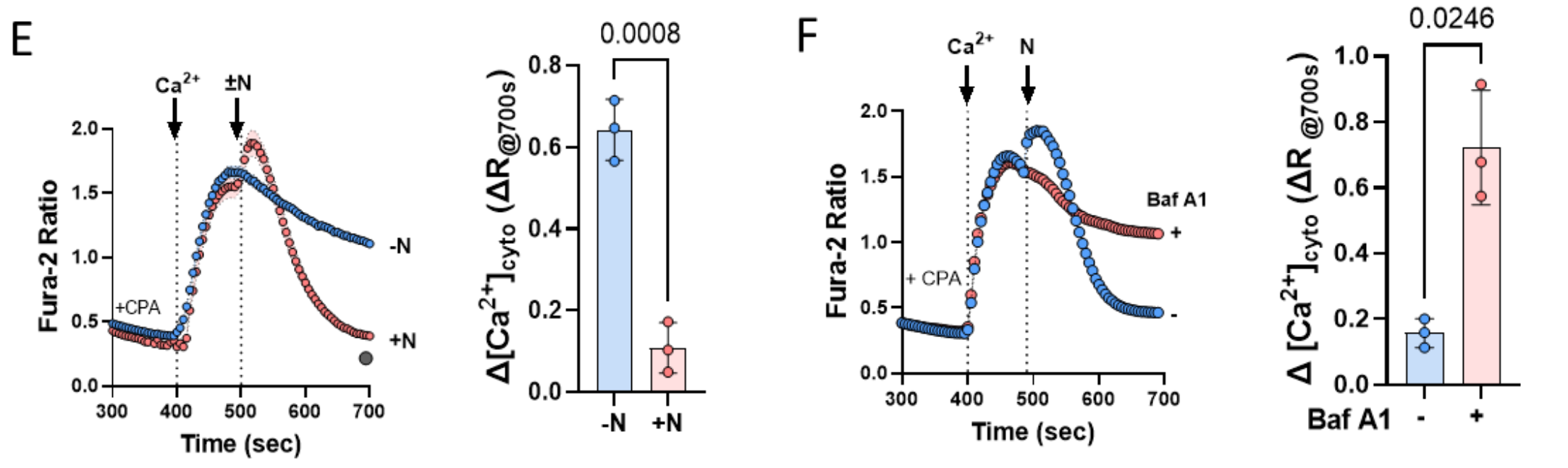

Figure 4E and 4F in the revised manuscript

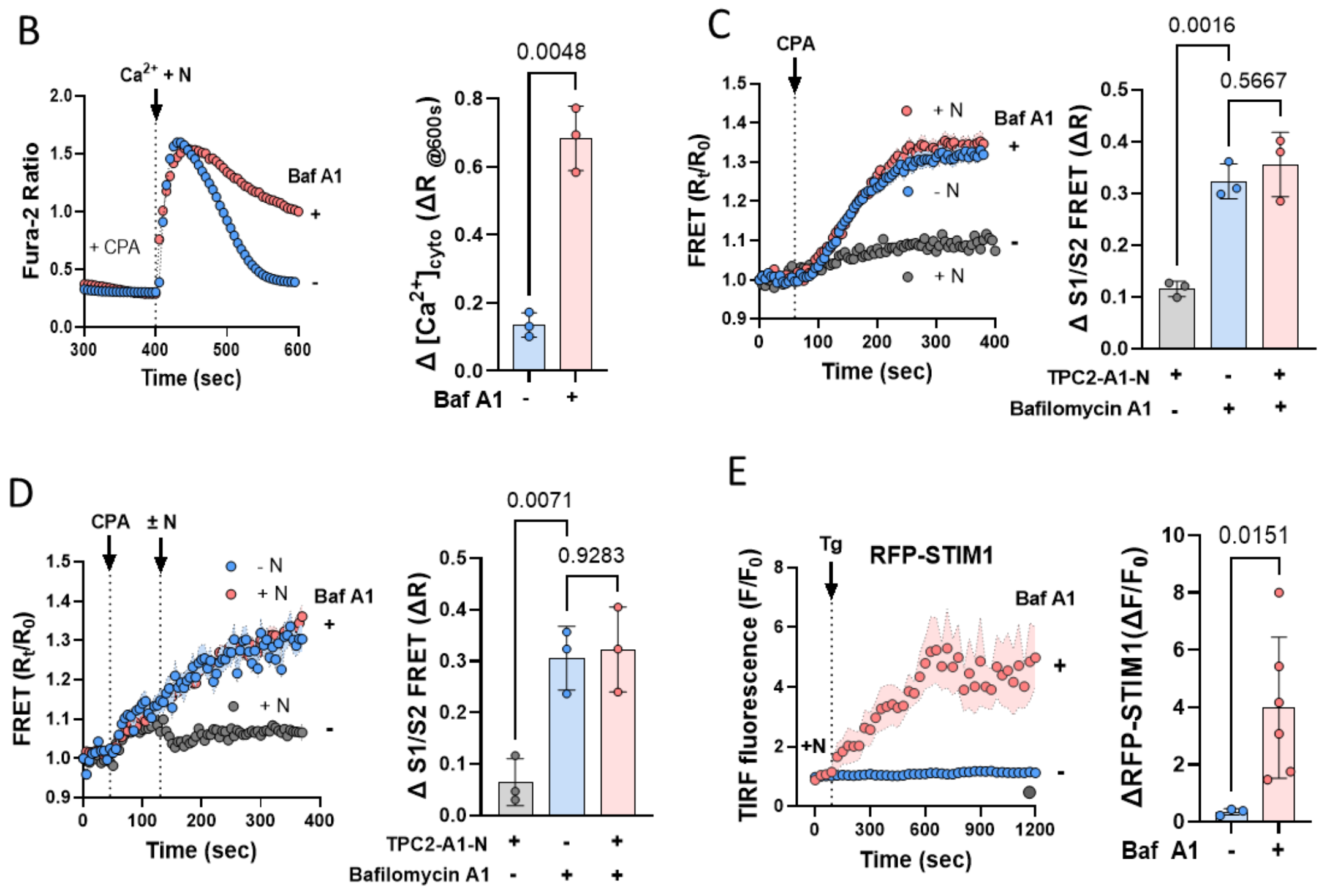

Figure S5B-E in the revised manuscript

Minor.

Figure 1D. This shows nicely that the TPC2 pore mutants act as dominant negatives on bulk calcium. But it is less clear what overexpression of TPC2 does here as there is no appropriate control (inert over-expressed protein/mock transfection). Previous work suggested that overexpressing TPC2 increases the rate of Ca2+ release by N not the amplitude (ref 14).

[Answer] We agree with the reviewers' comment, and the revised data includes TPC2-A1-N–evoked responses in cells transfected with the empty vector (pcDNA) (Fig. 1H).

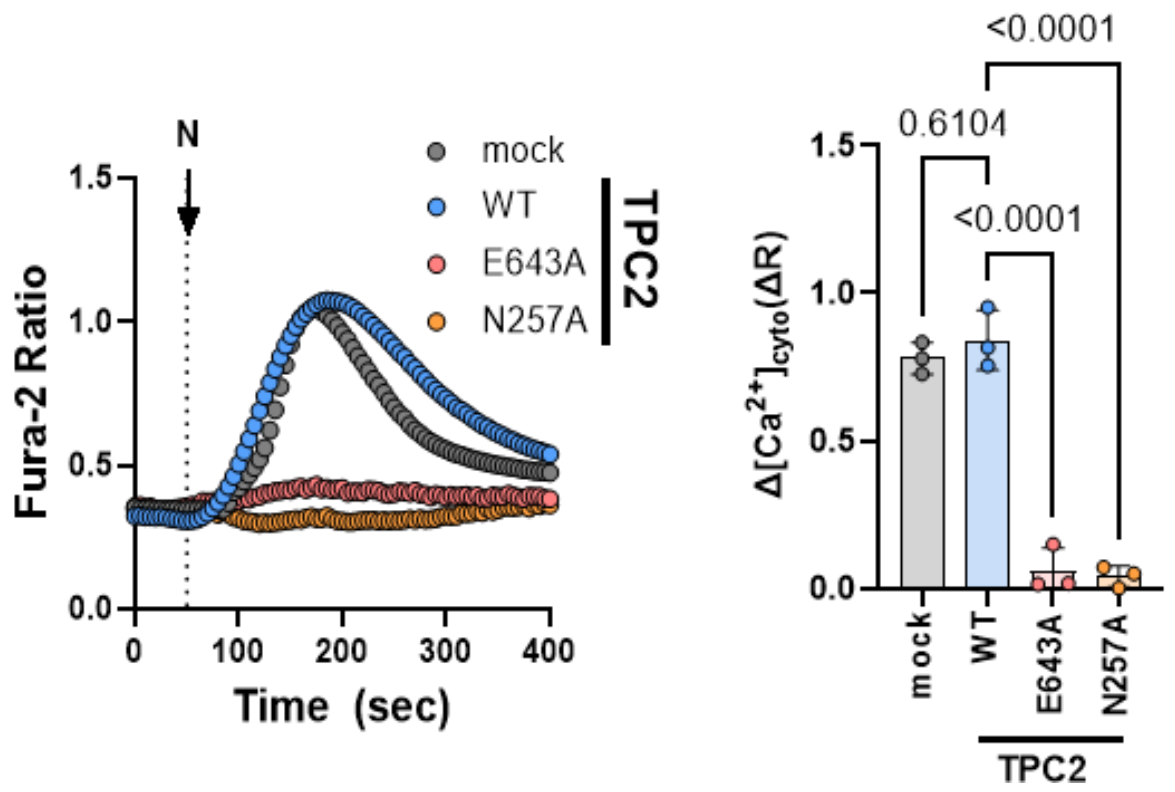

Figure 1H in the revised manuscript

Fig. 1 Legend. It is stated 'Data are mean ±SD of N = 3-4 independent recordings from 130/101 cells (B), 171/174, 106/102, and 61/55 cells (C-E), 189/196, 121/100, and 58/55 cells (F-H), two-tailed unpaired t test with Welch's correction' What does '130/101' mean? Number of cells from 2 experiments? But it is stated that data are from 3-4 experiments. If these experiments are truly independent then perhaps s.e.m should be used. Please clarify, maybe as a section in the methods.

[Answer] The large numbers are the total number of cells recorded for each condition in the bar graphs. '130/101' indicates that a total of 130 cells treated with Tg and 101 cells treated with A1-N were recorded in 3 independent experiments in Fig. 1B. Dots show values from individual experiments, bars their average with SD. We added this explanation to the figure legends.

Figure 2A. The author have succeeded in picking up endogenous TPC2 by Western blotting (something the reviewer' s lab struggles with!). It would be nice to see what siRNA (Fig. S1) does here if the authors decide on retaining these data (see major point 2).

[Answer] As discussed above, we have removed these data and plan to clarify the relative contribution of RyR and IP3R to the ER Ca^{2+} release evoked by TPC2-A1-N in a subsequent study. For detection of endogenous TPC2 by Western blotting, we used an anti-TPC2 antibody purchased from Alomone labs. Although siTPC2 knockdown reduced TPC2 protein levels (Fig. R3 below), we decided not to include this result in the revised manuscript.

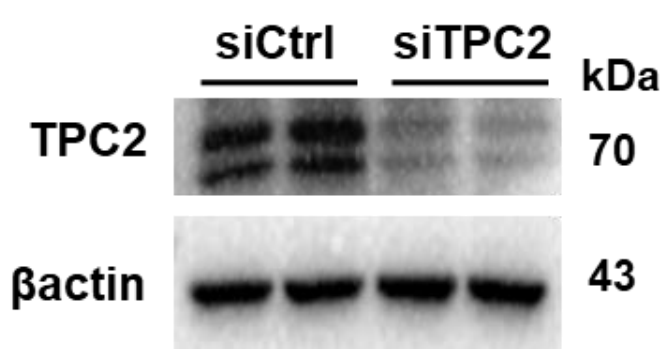

Figure R3. Western blot analysis shows reduced TPC2 protein levels following siTPC2-mediated silencing in HEK cells.

Fig.3F. Please add a control for TPC2 expression here so we can see the extent of reversal by the TPC2 mutants.

[Answer] We added the pcDNA(empty vector)-transfected group to the graph illustrating TPC2-A1-N–induced SOCE inhibition, which is now in Fig. 2F in the revised manuscript.

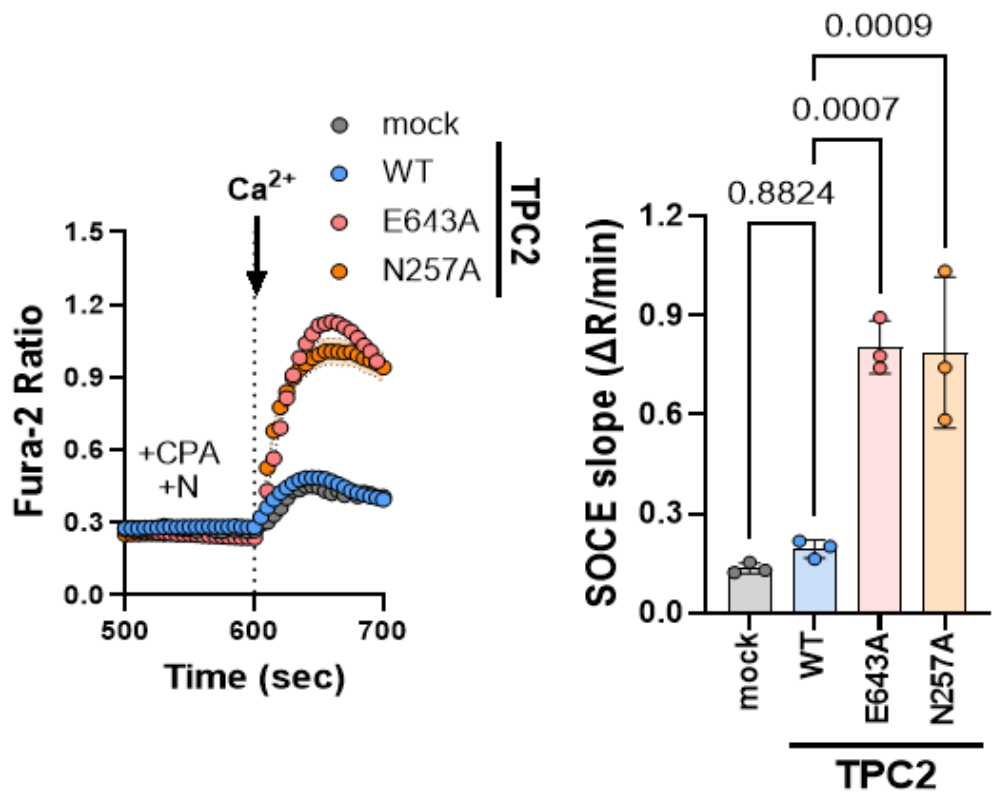

Figure 2F in the revised manuscript

Fig. S3A. The finding that bafilomycin reverses N block is important. Please bring this into the main Figure perhaps by replacing the dantrolene data, given my concerns above about ryanodine receptors.

[Answer] As suggested, we have moved the bafilomycin data to the main figures (Fig. 2G) and removed the dantrolene data.

Figure 4. It would be nice to see some negative controls here on CRAC currents. Can the authors test the effects of TPC2-A1-P (major point 1) and NADP?

[Answer] Fig S3C now shows the effect of NADP on CRAC currents. We did not record CRAC currents in the presence of TPC2-A1-P as very high concentrations would be required for inhibition.

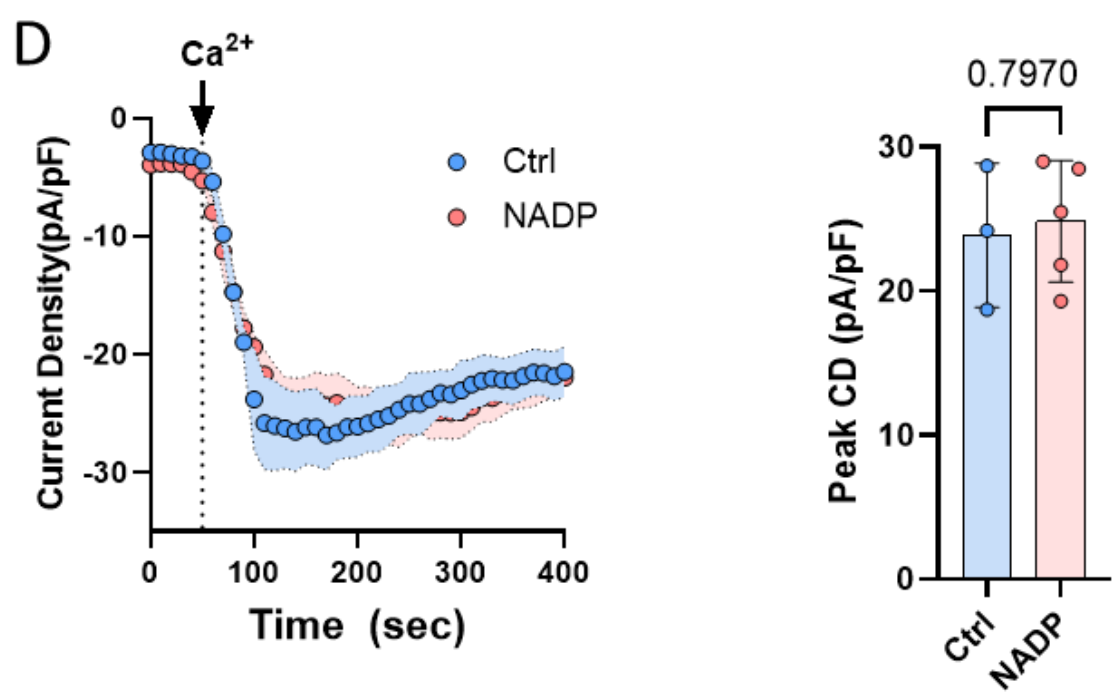

Figure S3C in the revised manuscript

Figure 5. As the authors quite rightly state, TPC2 activation 'remarkably' inhibits on going SOCE. it would be prudent to test specificity of the compound here using some of the strategies applied earlier. This is because it is difficult to explain at present how a presumed local calcium signal can have a functional effect in the face of global calcium influx.

[Answer] We thank the reviewer for this suggestion. Fig 4F and S5B now show that bafilomycin completely prevents the acute inhibition of ongoing SOCE by A1-N. In addition, Fig S4C and S4E shows that bafilomycin relieves the TPC2-dependent dissociation of STIM1 and its retrieval from the PM by FRET and TIRF.

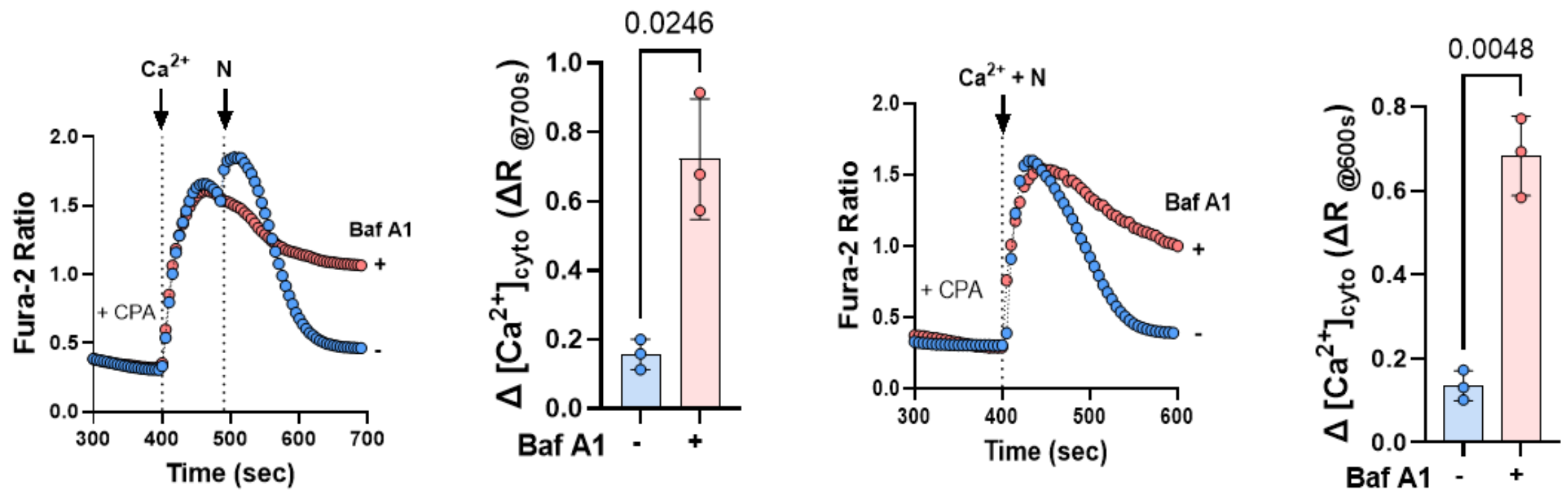

Figure 4F and S4B in the revised manuscript

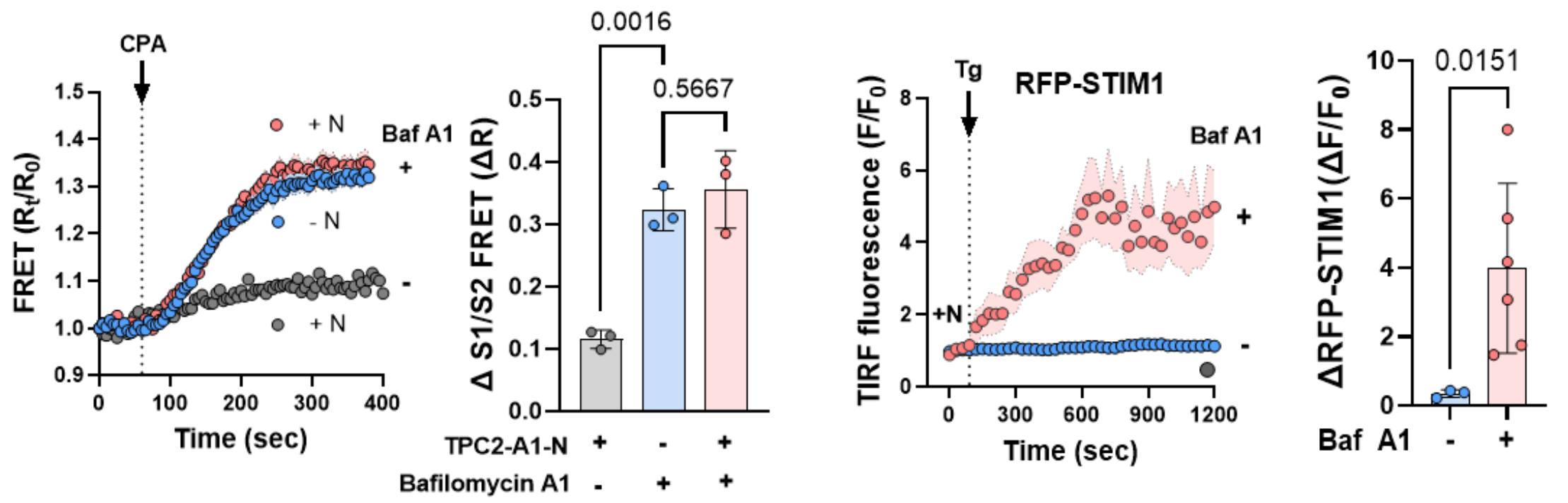

Figure S4C and S4E in the revised manuscript

Figure 7. Here, Jurkat cells are pretreated with N and this is shown to block physiological Ca2+ responses to T cell activation. Can we please see what N does acutely as supp data? I ask because there have been unpublished reports at scientific meetings showing that N induces robust non-specific Ca2+ entry in these cells.

[Answer] We have performed the suggested experiment. The fura-2 responses evoked by 20 µM TPC2-A1-N in adherent Jurkat T cells kept in Ca^{2+}-rich saline were complex (Fig. R4 below). Three phases could be distinguished: an initial transient elevation (1), probably reflecting Ca^{2+} release from lysosomal and ER stores, followed by a decline that was more pronounced in cells with high basal $[Ca^{2+}]_{cyt}$ levels (2) and, in a fraction of cells, a delayed and sustained $[Ca^{2+}]_{cyt}$ elevation (3). The fraction of cells undergoing secondary elevations varied between 7 and 60% between independent experiments (Fig. R3, right panel). Given the complexity and variability of these responses and the debated role of TPC2 channels in T lymphocytes, we prefer to abstain from presenting these data and plan to characterize the effects of TPC2-A1-N in detail in a subsequent study.

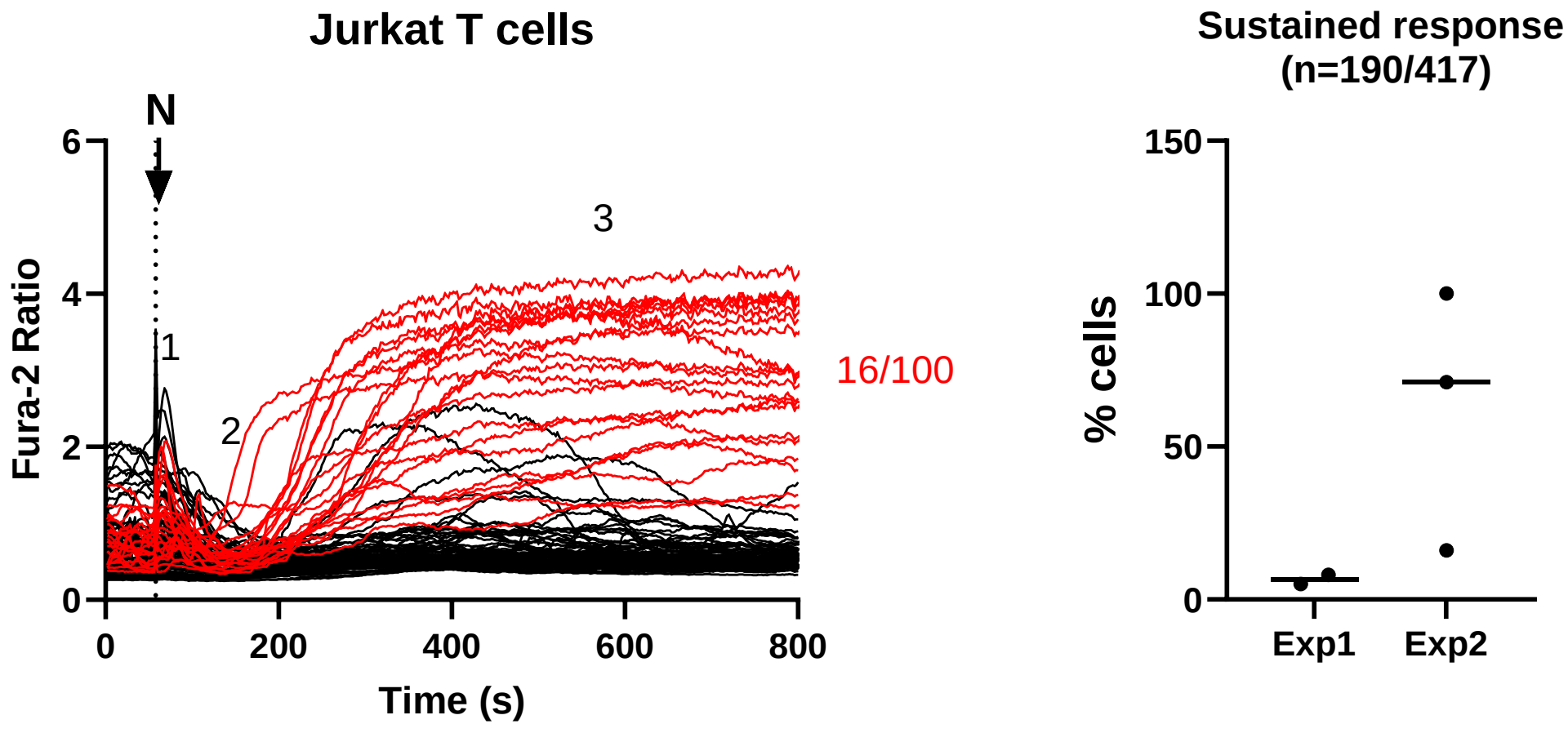

Figure R4. Left: representative Fura-2 responses evoked by TPC2-A1-N (20 µM) in Jurkat T cells in Ca^{2+}-rich medium. N=100 cells. Right: Fraction of cells exhibiting a delayed sustained $[Ca^{2+}]_{cyt}$ response. N= 5 recordings of 22/88/64/143/100 cells in 2 independent experiments.

Discussion: The authors should discuss this paper as it is very relevant to some of the effects reported here: *https://pubmed.ncbi.nlm.nih.gov/33328326/*

[Answer] The indicated paper is now cited. We have contacted the authors and apologized for the oversight.

Really minor!

Abstract: It is stated 'The TPC2 agonists NAADP and TPC2-A1-N...' I suggest using 'activators' to make a subtle distinction between the two given that NAADP acts indirectly through NAADP binding proteins. Done.

Introduction: Please reference this sentence: 'TPC2 is a dual-gated ion channel of late endosomes and lysosomes comprising two homologous domains that dimerize to form a functional "quasitetrameric" channel whose selectivity is agonist-dependent, switching between Na+ and Ca2+ based on the stimulus.' We now refer to the Yuan et al. Nat Commun 2022 paper as source for this sentence.

Introduction: Consider deleting the following sentence as the authors of the referenced work find no role for TPC2 in T cells: 'In T-cells, JPT2 coprecipitates with type I ryanodine receptor (RyR) on the ER and with T cell receptors on the cell membrane and was proposed to coordinate the NAADP-mediated activation of lysosomal TPC2 and ER RyR channels that generate Ca2+ microdomains during the first seconds of T cell activation 28, 31. TPC2-RyR coupling amplifies NAADP-mediated Ca2+ signals in T-cells..' Done.

Introduction: Please fix:'The later inactivation constrain..'. Fixed

Results: Please fix repetition in: 'Furthermore, BAPTA-AM prevented the acute inhibition of STIM1 oligomerization evoked by TPC2-A1-N observed in Fig. 5C abrogated in the presence of BAPTA-AM (Fig. 6E).' Fixed

Discussion: Fix: ' PI(3,5)P3' Fixed. Thanks to reviewer for all these corrections.
